# Sounds of the northern Andes: the calls of a diverse and endangered frog community (Amphibia, Anura) from Ecuador

**DOI:** 10.3897/zookeys.1224.137972

**Published:** 2025-01-30

**Authors:** Diego Batallas, Rafael Márquez, Juan M. Guayasamin

**Affiliations:** 1 Departamento de Biodiversidad, Ecología y Evolución, Facultad de Ciencias Biológicas, Universidad Complutense de Madrid, Calle J.A. Nováis 12, 28040, Madrid, Spain Universidad Complutense de Madrid Madrid Spain; 2 Laboratorio de Biología Evolutiva, Colegio de Ciencias Biológicas y Ambientales COCIBA, Universidad San Francisco de Quito USFQ, vía Interoceánica y Diego de Robles 17-1200-841, Quito, Ecuador Universidad San Francisco de Quito Quito Ecuador; 3 Instituto Nacional de Biodiversidad (INABIO), Calle Rumipamba 341 y Av. de Los Shyris, Casilla Postal 17-07-8976, Quito, Ecuador Instituto Nacional de Biodiversidad Quito Ecuador; 4 Fonoteca Zoológica, Departamento de Biodiversidad y Biología Evolutiva, Museo Nacional de Ciencias Naturales (CSIC), 28006, Madrid, Spain Museo Nacional de Ciencias Naturales Madrid Spain

**Keywords:** Andean mountains, anurofauna, bioacoustics, Carchi province, conservation

## Abstract

The emission of calls is one of the most distinctive and important reproductive traits in anurans. Given the biological significance of vocalizations, this trait is also useful for identification proposes and is key in recognizing cryptic diversity. However, the majority of the calls from tropical ecosystems, especially in the high Andean mountains, are unknown. Between 2016 and 2021, a total of 14 expeditions were conducted to the forests and moorlands of the eastern and western Andean Mountain range of the province of Carchi-Ecuador, at elevations ranging from 2694 to 3848 m a.s.l. The objective of these expeditions was to record the calls of the anuran fauna present in these ecosystems. In total, 30 anuran species were recorded, and calls of 20 species were described, 15 of which are described and reported for the first time in the present study. The call of *Hyloxalusdelatorreae*, a critically endangered species, is described with a remarkable recording of the call of *Niceforoniabrunnea*, a species considered mute. In addition, nine are candidate species, including the first record of *Pristimantisfarisorum* for Ecuador. This study represents the most comprehensive and accurate acoustic documentation of a highland community, which will facilitate taxonomic and conservation work in the area.

## ﻿Introduction

Acoustic communication is one of the most varied communication systems that animals use to transmit information and interact with each other (Redondo 1994; Bradbury and Vehrencamp 1998; Simmons 2003). This type of communication is one of the most distinctive, important, and conspicuous ethological traits of anurans (Zelick et al. 1999; Colafrancesco and Gridi-Papp 2016). They emit different types of calls, which are associated with a specific social context and function (Wells and Schwartz 2007; Toledo et al. 2015; Köhler et al. 2017). Given the evolutionary role of vocalization in conspecific recognition, the knowledge and analysis of anuran calls represent a relevant tool for species identification, especially in sympatry. The analysis and determination of call variations, in conjunction with the use of molecular and morphometric tools, has enabled the resolution of phylogenetic and taxonomic inconsistencies in certain groups of species. (e.g., Páez-Vacas et al. 2010; Hutter and Guayasamin 2015; Caminer et al. 2017; Ron et al. 2018; Páez and Ron 2019; Yánez-Muñoz et al. 2021). The study of species using integrative approaches has led to a more comprehensive understanding of diversity in taxonomic, phylogeographic, evolutionary, and conservation terms (Angulo and Reichle. 2008; Gill et al. 2016; Mendoza-Henao et al. 2020, 2023).

This study focuses on the acoustic description of several localities in the Andes of northern Ecuador, part of the tropical Andes, one of the most biodiverse regions on Earth (Duellman 1999; Hutter et al. 2017; Hazzi et al. 2018). The northern Andes exhibit the highest levels of endemism among ecosystems (Armesto and Señaris 2017; Guayasamin et al. 2020; Yánez-Muñoz et al. 2020). However, the region is threatened by the constant destruction of its habitat (Guayasamin et al. 2022). As a consequence, a significant part of the biodiversity present in these ecosystems being in critical danger of extinction (Ojala-Barbour et al. 2019; Yánez-Muñoz et al. 2020).

In this study, we characterized and described the acoustic parameters of anurans calls present in the high Andean ecosystems of northern Ecuador, Carchi province. We highlight the utility of vocalizations to identify species and to discover new taxa.

## Materials and methods

### Study area

**Figure 1.**
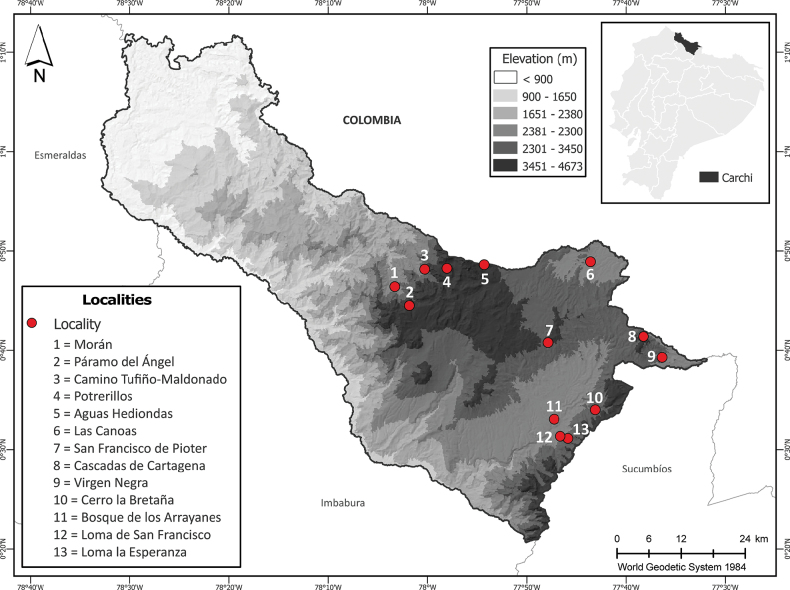
Map of Carchi province, Ecuador, showing the exact locations of the sampled localities.

**Figure 2.**
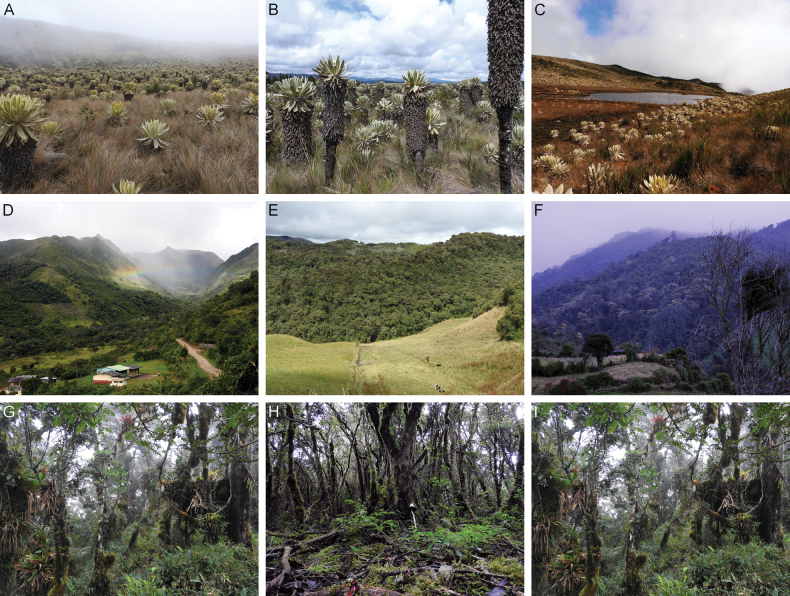
Characteristic habitats of the high-altitude Andean ecosystems of northern Ecuador, Carchi Province. **A** reserva ecológica del Ángel **B** Páramo San francisco de Pioter **C** Laguna cerro la Bretaña **D** Morán **E** Virgen Negra **F** San Francisco de Pioter **G** Cerro la Bretaña **H** La Changadera-Morán; **I**) San francisco de Pioter; Bosque de los Arrayanes. The upper part shows the Páramo ecosystems, the middle part a general overview, and the lower part the Andean montane forests.

A total of 13 localities were selected in the northern Andes of Ecuador, Carchi province, at elevations ranging from 2694 to 3848 m a.s.l. (Table 1). The habitats and microhabitats of the different high Andean ecosystems were examined, encompassing the western and eastern slopes of the Andes (Figs 1, 2).

**Table 1.** Details of the 11 localities and ecosystems of the province of Carchi (Ecuador) that were sampled in this study. The abbreviations used in the ecosystem correspond to: Northeastern Andean High Mountain Evergreen Forest (**BsAn01**); Northwestern Andean High Mountain Evergreen Forest (**BsAn03**); Northwestern Andean Montane Evergreen Forest (**BsMn03**); Northeastern Andean Montane Evergreen Forest (**BsMn01**); Paramo Evergreen Forest (**BsSn01**); Caulescent Rosette and Páramo Grassland (frailejones) (**RsSn01**). The classification system proposed by the Ministerio del Ambiente (2013) is followed.

Locality	Coordinates	Altitude	Ecosystem
Moran	0°46'07.10"N, 78°03'20.00"W	2785	BsMn03
Bosque de los Arrayanes	0°33'03.44"N, 77°47'14.68"W	2870	BsMn01
Las canoas	0°48'55.2"N, 77°43'34.4"W	2877	BsMn03
Loma San Francisco	0°31'20.7"N, 77°46'38.1"W	2922	BsAn01
Cascadas de Cartagena	0°41'25.3"N, 77°38'14.1"W	3068	BsAn01
Loma la Esperanza	0°30'57.6"N, 77°46'09.5"W	3118	BsAn01
Cerro la Bretaña	0°34'07.92"N, 77°42'53.00"W	3226	BsAn01
Camino Tufiño-Maldonado	0°48'10.0"N, 78°00'16.8"W	3362	BsMn03
San Francisco de Pioter	0°40'16.1"N, 77°47'43.9"W	3416	BsAn03
Aguas hediondas	0°48'38.01"N, 77°54'15.5"W	3595	BsSn01
Potrerillos	0°48'15.5"N, 77°58'03.1"W	3785	BsSn01
Páramo del Ángel	0°44'29.3"N, 78°01'49.3"W	3848	RsSn01
Virgen Negra	0°39'17.57"N, 77°36'21.13"W; 0°39'54.2"N, 77°38'42.1"W	2994 3627	BsAn01 RsSn01

### Field techniques

A total of 14 expeditions were conducted across two distinct phases. The initial phase of the study was conducted between December 2016 and May 2017 and comprised of a total of six expeditions, with ten sampling days per month. The second phase between 2020–2021 comprised eight field expeditions (five in 2020 and three in 2021), each of which lasted 12 days (limited by the restrictions imposed by the Covid-19 pandemic). The rainfall regime of the northern zone of Ecuador, from October to May, was considered in the context of the fieldwork. At the selected localities, individualized recordings were made utilizing direct visual encounters and auditive bands transects (Heyer et al. 2014; Rueda-Almondacid et al. 2006) between 18:00 and 23:00 h; similarly, recordings were made between 5:00 and 10:00 h and 15:00 and 17:00 h, in order to record anurans exhibiting nocturnal, diurnal, and crepuscular activities, respectively.

The calls were recorded using an Olympus LS-100 digital recorder, which was coupled to a Sennheiser K6-C modular system and ME 66 shotgun microphone head, or to a Rode NTG3 shotgun microphone. All recordings were made at a sampling rate of 44.1 kHz and 16 “bits” resolution, saving the audio files in the uncompressed WAV format. Air temperature and humidity data for each of the recordings were taken with a Taylor 1523 digital thermohydrometer. After recording, specimens were manually located and collected. Specimens were sacrificed according to the recommendations of Chen and Combs (1999) and preserved according to the protocols of Simmons (2015), with liver samples extracted for genetic analyses and preserved in 99% ethanol. The handling and collection of specimens was conducted under permits granted by The Ministerio del Ambiente y Agua de Ecuador: MAE-DNB-CM-2016-0045 y MAE-DNB-CM-2018-0105.

The specimens were deposited at the División de Herpetología del Instituto Nacional de Biodiversidad (**DHMECN**), Quito, Ecuador and at the Museo de Zoología de la Universidad San Francisco de Quito (**ZSFQ**). Tissues for molecular analyses are deposited at the Laboratorio de Biología Evolutiva de la Universidad San Francisco de Quito (**LBE**) and acoustic recordings are deposited at the Fonoteca Zoológica (www.fonozoo.com) del Museo Nacional de Ciencias Naturales (**CSIC**), Madrid, Spain.

### Bioacoustic analysis

**Figure 3.**
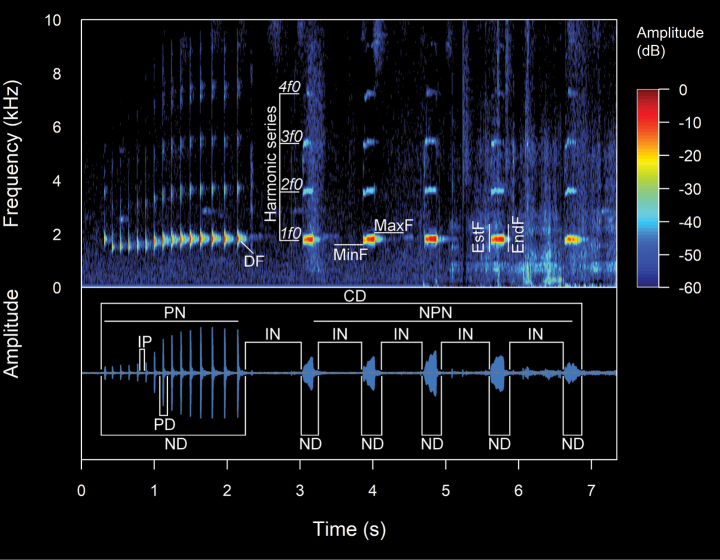
The spectral (above) and temporal (bellow) parameters used in the analyses performed in this study. Abbreviations: **CD** Call duration; **ND** Note Duration; **PN** Pulsed note; **NPN** Non-Pulsed Notes **IN** Intervals between notes; **PD** Pulse duration; **IP** Intervals between pulses; **DF** Dominant frequency; **MinF** Minimum frequency; **MaxF** Maximum frequency; **Estf** Start-frequency; **Endf** End-frequency; **Harmonic Series** series of harmonics visible in the frequency spectrum (1f0 = first harmonic, 2f0 = second harmonic, 3f0 = third harmonic, 4f0 = fourth harmonic)

**Figure 4.**
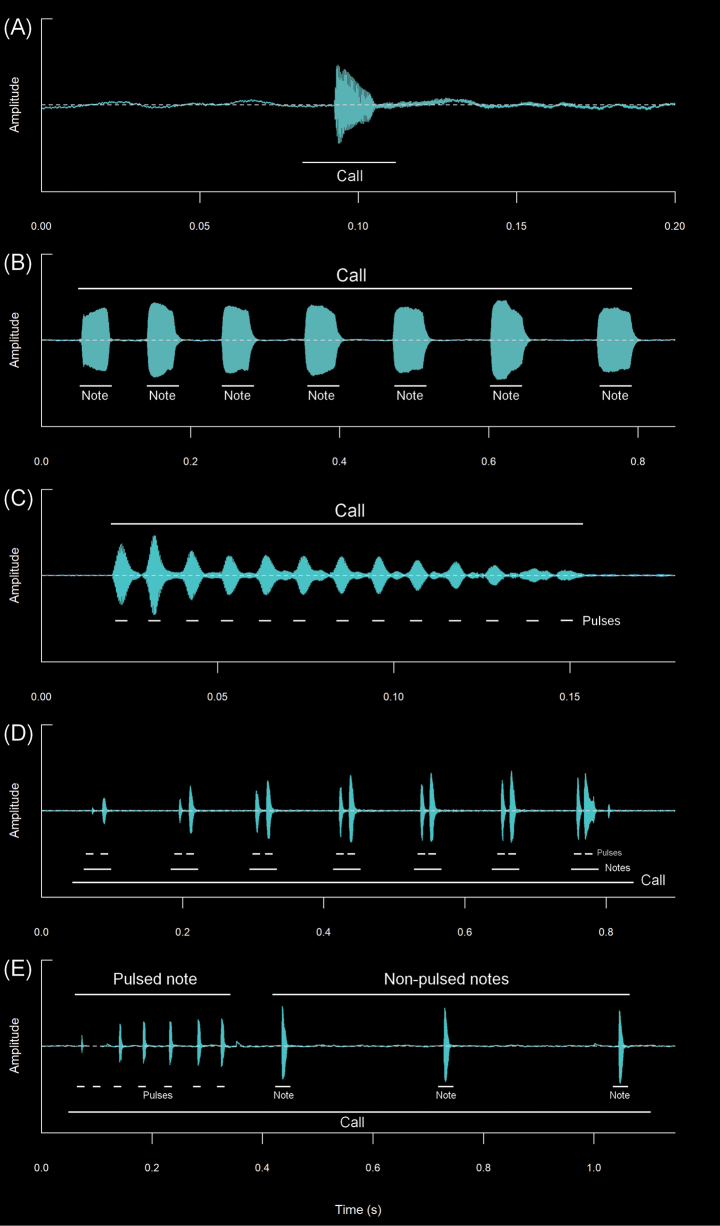
Types of calls analyzed in this study, classified based on their temporal characteristics and note-centered focus (Köhler et al. 2017; Emmrich et al. 2020). **A** non-pulsed simple call (*Pristimantis* sp. 4; *Pristimantismyersi*; ZSFQ 4427) **B** call with uniform non-pulsed notes (*Hyloxalus* sp.; ZSFQ 4442) **C** call with one pulsed note (*Centrolenebuckleyi*; DHMECN 13375) **D** call with uniform pulsed notes (*Pristimantis* sp. 5; *Pristimantismyersi* group; DHMECN 13334) **E** complex call (*Pristimantis* sp. 3; *Pristimantismyersi*; ZSFQ 4554).

The spectral and temporal parameters of calls were analyzed using the software Raven 1.6 (K. Lisa Yang Center for Conservation Bioacoustics 2024), using for the spectrograms Hann window with 256 samples of the Fast Fourier Transformation (FFT), and time grids with a hop size of 26 samples, with 90% of overlap and a frequency grid with 512 samples of the Discrete Fourier Transformation (DFT), and a 3 dB filter bandwidth of 248 Hz. The parameters analyzed were: (**CD**) Call duration: Total time elapsed from the beginning to the end of a call. (**IC**) Interval between calls: Duration of the interval of silence between two consecutive calls. (**CR**) Call rate: Total number of calls emitted in a specific period of time, calculated as calls per minute. In this study, the call rate was determined by analyzing the entire recording, which contained regular sequences of calls while avoiding intervals with very prolonged silences. **(NC)** Notes/call: Number of notes in a call, where a note is a subunit of a call separated from other notes by intervals of silence. (**ND**) Note Duration: Total time elapsed from the beginning to the end of a note. (**IN**) Intervals between notes: Duration of the interval of silence between two consecutive notes. (**NR**) Note rate: Total number of notes emitted in a specific period of time, calculated as notes per second. (**PN**) Pulse/note: Number of pulses in a call or note, where a pulse is the shortest indivisible subunit of a call. (**PD**) Pulse duration: Total time elapsed from the beginning to the end of a pulse. (**IP**) Intervals between pulses: Duration of the interval of silence between two consecutive pulses. (**PR**) Pulse rate: Total number of pulses emitted in a specific period of time, calculated as pulses per second. (**FF**) Fundamental frequency: The lowest frequency or first harmonic of a harmonic series, which frequently coincides with the dominant frequency. (**DF**) Dominant frequency: The frequency that contains the highest energy within the call or the peak of the frequency spectrum with the highest amplitude value. (**MinF**) Minimum frequency: Minimum or lower limit of the frequency, with consideration given to the frequency at 5% for this value. (**MaxF**) Maximum frequency: Maximum or upper limit of the frequency, with consideration given to the frequency at 95% for this value. (**FM**) Frequency modulation: Variation of the dominant frequency that increases or decreases in comparison to the initial and final frequencies of the call. (**NH**) Number of visible harmonics: Definitions, terminology, and measurements of acoustic parameters have been reviewed and adapted from the works of Cocroft and Ryan (1995), Köhler et al. (2017), and Sueur (2018). The definition of call structure (note-pulse) and the calculation of frequency modulation were based on Emmrich et al. (2020). In the measurement of the minimum and maximum frequencies we followed the recommendations of Forti et al. (2019). The variability of the calls generated different types of interpretations and values for their temporal and spectral parameters (see Figs 3, 4). The oscillogram and spectrogram figures were processed and constructed in the R software (R Development Core Team 2024), through the Seewave package v. 2.1.4 (Sueur et al. 2008), using a Hann window at 99% overlap with a size of 256 samples of the fast Fourier transform (FFT). The audio files in WAV format were imported with the tuneR package v. 1.4.7 (Ligges et al. 2018). With the values of the analyzed parameters, the measures of central tendency (means) and dispersion (maximum, minimum, and standard deviation) were calculated. A Principal Component Analysis (PCA) was conducted using the prcomp function in R software (R Development Core Team 2024) leveraging the mean values of eight acoustic variables from the analyzed calls to preliminarily and prospectively determine the acoustic differentiation among the species under study. The results of the PCA were subsequently visualized using the ggplot2 package in R (Wickham 2016).

### Taxonomic identification and candidate species

Taxonomic nomenclature follows Frost (2024). The identification of the collected males was conducted using a combination of specialist keys, reference materials, taxonomic reviews, museum collections, and input from subject matter experts (e.g., Coloma 1995; Lynch and Duellman 1997; Duellman and Lehr 2009; Duellman 2015; Guayasamin et al. 2020). In regard to the candidate species, we have adhered to the terminology and designations set forth by Franco-Mena et al. (2023). These authors, in turn, followed Vieites et al. (2009) and Padial et al. (2010), particularly in the case of the *Pristimantismyersi* group. The molecular revisions and sequencing of this group were conducted using genetic material derived from the collections generated in the present study. The remaining species, for which no specific identity has been determined, are regarded as unconfirmed candidates based on their morphological and bioacoustical characteristics.

## Results

**Plate 1.**
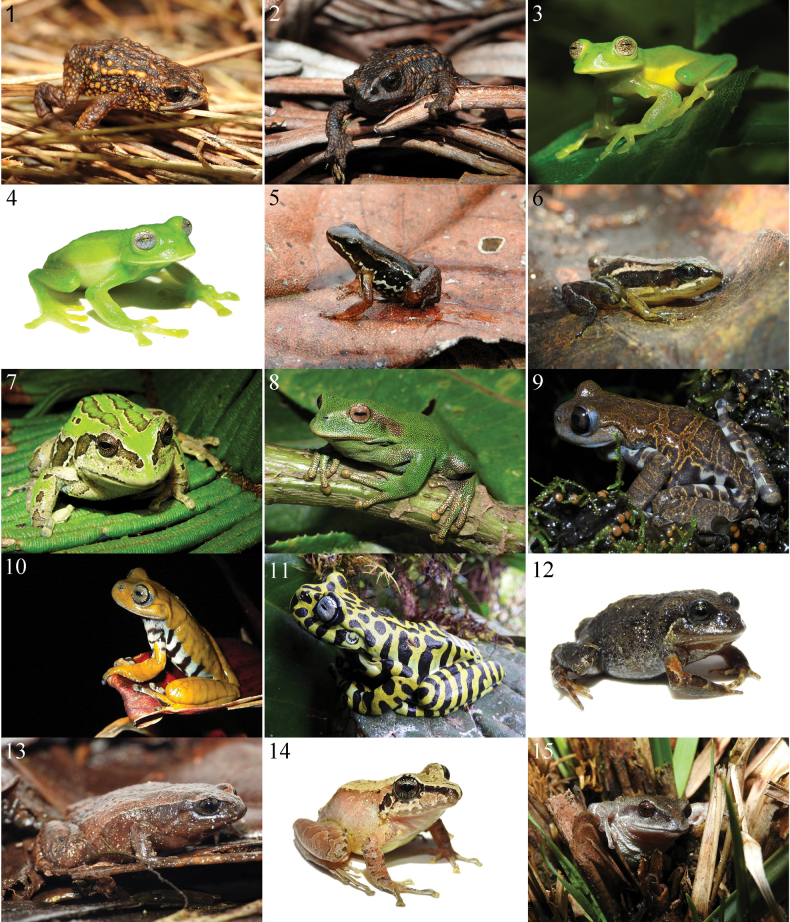
The anurans of the high Andean ecosystems of the Carchi province in Ecuador. **1***Osornophryneangel*DHMECN 13783 **2***Osornophrynebufoniformis*DHMECN 13763 **3***Centrolenebuckleyi*ZSFQ 4421 **4***Nymphargus* sp. ZSFQ 6778 **5***Hyloxalusdelatorrae* (not_collected) **6***Hyloxalus* sp. ZSFQ 4442 **7***Gastrothecaespeletia*DHMECN 13758 **8***Gastrothecaorophylax*DHMECN 13761 **9***Hyloscirtuscriptico* (not collected Photo: Mario Yánez-Muñoz) **10***Hyloscirtuslarinopygion*DHMECN 3799 Photo Mario Yánez-Muñoz) **11***Hyloscirtustigrinus* (not collected Photo: Libardo Tello) **12***Niceforoniabrunnea*ZSFQ 4470 **13***Noblella* sp. ZSFQ 4543 **14***Pristimantisactites*ZSFQ 4487 **15***Pristimantisbuckleyi*DHMECN 13670.

**Plate 2.**
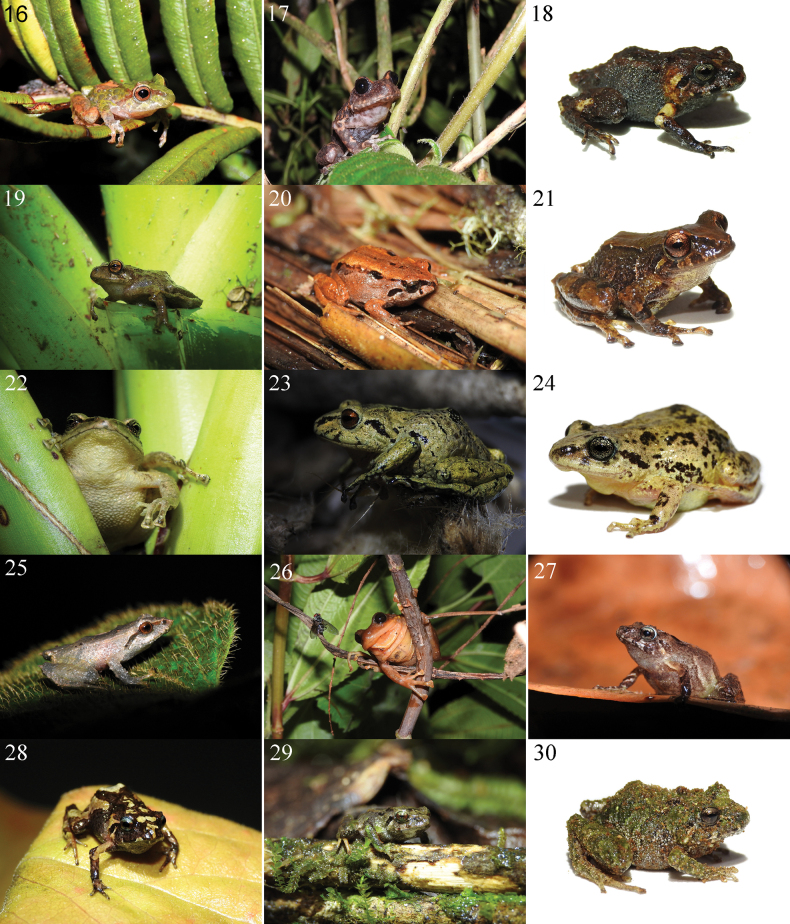
The anurans of the high Andean ecosystems of the Carchi province in Ecuador. **16***PristimantisChloronotus* DSCN6763 **17***Pristimantisfarisorum*DHMECN 13760 **18***Pristimantisfestae*ZSFQ 4438 **19***Pristimantishuicundo*DHMECN 13777 **20***Pristimantisocreatus*DHMECN 13650 **21***Pristimantispteridophilus*ZSFQ 4490 **22***Pristimantissupernatis*DHMECN 13759 **23***Pristimantisthymelensis*DHMECN 13787 **24***Pristimantisunistrigatus*DHMECN 13351 **25***Pristimantis* sp. (not collected) **26***Pristimantis* sp. 1 *ridens* group DHMECN 13753 **27***Pristimantis* sp. 2 *myersi* group DHMECN 13643 **28***Pristimantis* sp. 3 *myersi* group DHMECN 13775 **29***Pristimantis* sp. 4 *myersi* group DHMECN 13638 **30***Pristimantis* sp. 5 *myersi* group ZSFQ 4486.

**Figure 5.**
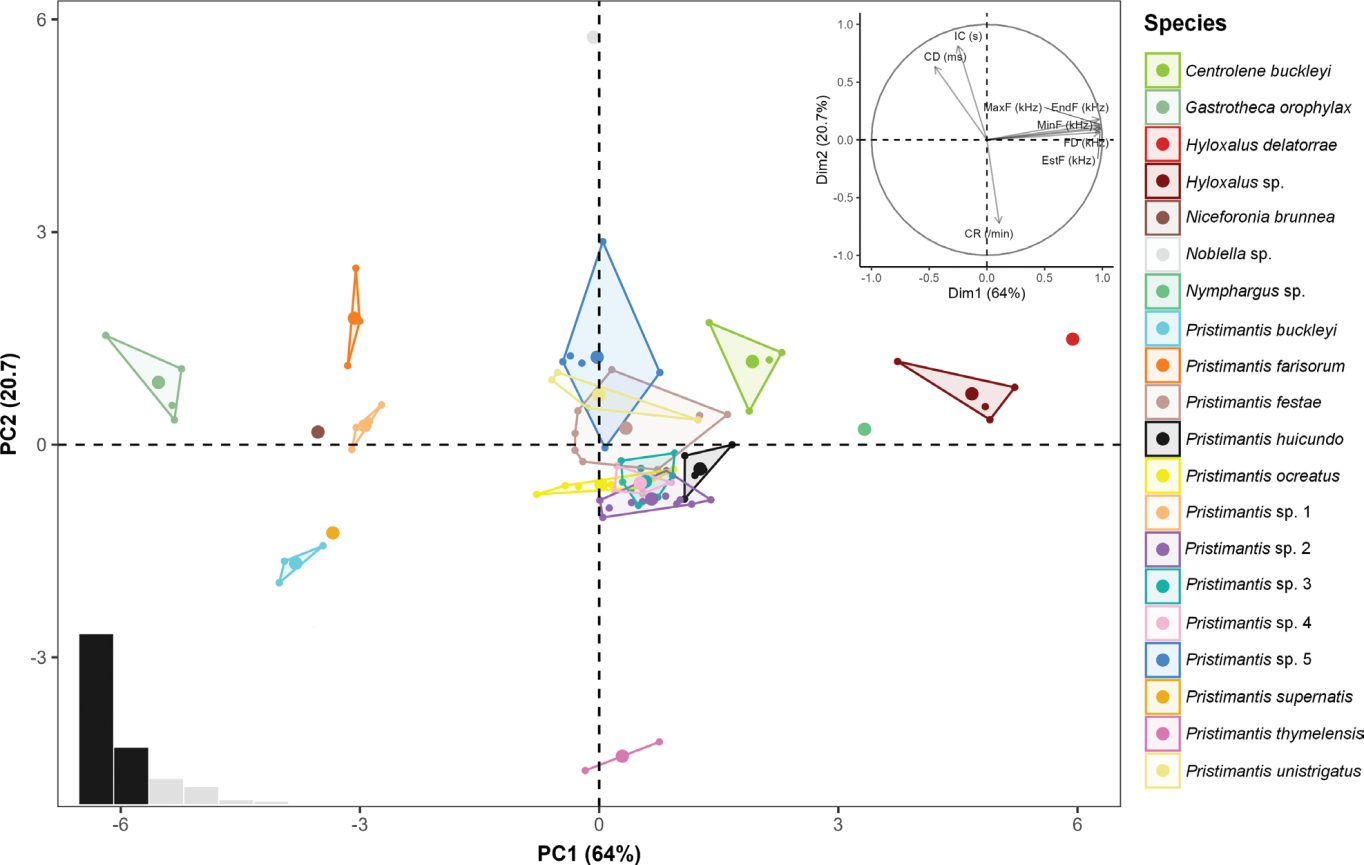
Principal Component Analysis (PCA) based on eight acoustic parameters of 20 Anuran species (Suppl. material 2, Table 2). The figure displays the number of principal components in the lower left-hand corner and the loading of each variable on the components in the upper right-hand corner.

A total of 30 species belonging to six families have been documented in the high-altitude Andean ecosystems of northern Ecuador, Carchi province (Plates 1, 2). From this diversity, recordings of 20 species were obtained by analyzing 1197 calls from 88 males (Suppl. material 2). Nine are candidate species, with the first record for Ecuador of *Pristimantisfarisorum* (Suppl. material 1, Table 2). The PCA of the calls of 88 males from 20 species showed eigenvalues > 1.0 in the first two components, which collectively account for 84.7% of the total variance. The first component (PC1) exhibits notable positive loadings on the spectral variables, including Dominant Frequency, Minimum Frequency, Maximum Frequency, Start Frequency, and End Frequency accounting for 64% of the variance. The second component (PC2) has significant negative loadings on the temporal variables, such as Call Duration, Interval Between Calls, and Call Rate (/min), accounting for 20.7% of the variance. The bioacoustics PCA demonstrates exploratory differences between the calls, manifesting as groupings and segregations among the various species, with some overlap observed in certain species groups (Table 3, Fig. 5). Below, we present detailed acoustic descriptions, organized by family:

**Table 2.** Anurofauna recorded in the high Andean ecosystems of northern Ecuador (Carchi province), with previous information on the knowledge of their calls. *Candidates species.

Family	Species	Sample analyzed		Advertisement call
		Males	Calls	
Bufonidae	*Osornophryneangel*	–	–	Not described
Bufonidae	*Osornophrynebufoniformis*	–	–	Not described
Centrolenidae	*Centrolenebuckleyi*	4	22	Bolívar et al. 1999; Guayasamin et al. 2006; Almendáriz and Batallas 2012; Guayasamin et al. 2020; Duarte-Marín et al. 2022; Cuellar-Valencia et al. 2023; Present study
Centrolenidae	*Nymphargus* sp.*	1	15	Present study
Dendrobatidae	*Hyloxalusdelatorrae*	1	10	Present study
Dendrobatidae	*Hyloxalus* sp.*	4	49	Present study
Hemiphactidae	*Gastrothecaespeletia*	–	–	Sinsch and Juraske 2006
Hemiphactidae	*Gastrothecaorophylax*	4	28	Sinsch and Juraske 2006; Present study
Hylidae	*Hyloscirtuscriptico*	–	–	Not described
Hylidae	*Hyloscirtuslarinopygion*	–	–	Rivera-Correa et al. 2017; Cuellar-Valencia et al. 2023
Hylidae	*Hyloscirtustigrinus*	–	–	Not described
Strabomantidae	*Niceforoniabrunnea*	2	5	Present study
Strabomantidae	*Noblella* sp.*	1	3	Present study
Strabomantidae	*Pristimantisactites*	–	–	Not described
Strabomantidae	*Pristimantisbuckleyi*	4	35	Cuellar-Valencia et al. 2023; Present study
Strabomantidae	*Pristimantischloronotus*	–	–	Not described
Strabomantidae	*Pristimantisfarisorum*	3	12	Present study
Strabomantidae	*Pristimantisfestae*	8	96	Holzheuser and Merino-Viteri 2019; Present study
Strabomantidae	*Pristimantishuicundo*	5	47	Present study
Strabomantidae	*Pristimantisocreatus*	6	103	Present study
Strabomantidae	*Pristimantispteridophilus*	–	–	Not described
Strabomantidae	*Pristimantissupernatis*	2	3	Present study
Strabomantidae	*Pristimantisthymelensis*	2	57	Present study
Strabomantidae	*Pristimantisunistrigatus*	4	22	Chaves-Acuña et al. 2023; Present study
Strabomantidae	*Pristimantis* sp. 1*	–	–	Not described
Strabomantidae	*Pristimantis* sp. 2 *ridens* group*	4	25	Present study
Strabomantidae	*Pristimantis* sp. 3 *myersi* group*	12	295	Present study
Strabomantidae	*Pristimantis* sp. 4 *myersi* group*	9	181	Present study
Strabomantidae	*Pristimantis* sp. 5 *myersi* group*	6	127	Present study
Strabomantidae	*Pristimantis* sp. 6 *myersi* group*	6	62	Present study

**Table 3.** Results obtained from Principal Component Analysis (PCA) of eight acoustic call variables from 20 frog species in Carchi, Ecuador.

	PC1	PC2
Dominant frequency	**0.992**	0.094
Minimum frequency	**0.99**	0.108
Maximum frequency	**0.987**	0.132
Start-frequency	**0.981**	0.066
End-frequency	**0.971**	0.178
Call duration	-0.45	**0.633**
Interval between calls	-0.251	**0.812**
Call Rate (/min)	0.107	-**0.721**
Eigenvalue	5.121	1.654
% Variance	64.009	20.676
% cumulative	64.009	84.685

The first component (PC1) exhibits notable positive loadings on the spectral variables, including Dominant Frequency, Minimum Frequency, Maximum Frequency, Start Frequency, and End Frequency, accounting for 64% of the variance. The second component (PC2) has significant negative loadings on the temporal variables, such as Call Duration, Interval Between Calls, and Call Rate (/min), accounting for 20.7% of the variance. The bioacoustics PCA demonstrates exploratory differences between the calls, manifesting as groupings and segregations among the various species, with some overlap observed in certain species groups (Table 3, Fig. 5). Below, we present detailed acoustic descriptions, organized by family.

### CENTROLENIDAE

#### *Centrolenebuckleyi* (Boulenger, 1882)

**Figure 6.**
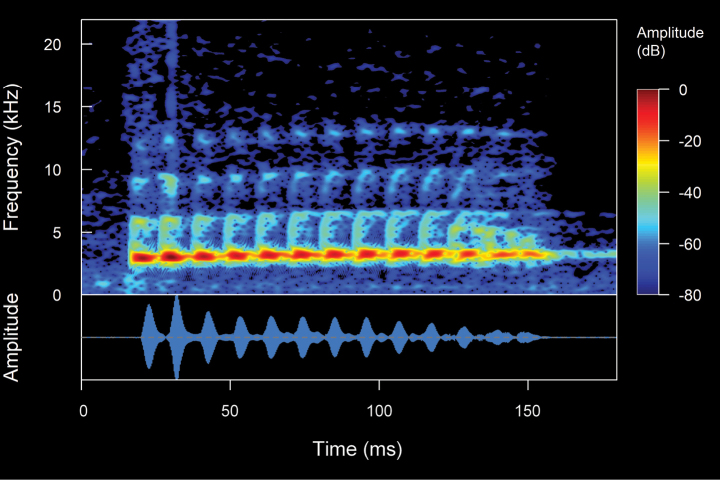
Spectrogram and oscillogram of the advertisement call of *Centrolenebuckleyi* (DHMECN 13375, SVL 29.37 mm, 10 °C air temperature, 86% relative humidity). Spectrogram obtained using the Hann window at 99% overlap, 256 samples of FFT size and 3 dB filter bandwidth of 248 Hz.

We recorded four males (Suppl. material 2, Table 2). The call (Fig. 6) is characterized by emission of a pulsed “Tri” type note (sensu Duarte-Marín et al. 2022). The recorded males were calling perched on herbaceous and shrubby vegetation in a marshy area ~ 50–150 cm above the ground. The calls are loud and can be clearly audible at distances of up to ca 200 m. *Centrolenebuckleyi* is a nocturnal species with moderate vocal activity, which intensifies following a drizzle. The mean call duration is 140.18 ± 16.35 ms (range 119–183), emitted at mean intervals of 20.88 ± 6.19 s (range 11.96–32.7 s), with a mean rate of 3.1 ± 0.92 calls/minute (range 1.83–4.96 calls/minute). The calls are composed of a mean of 12.32 ± 1.13 pulses (range 11–15 pulses). The mean pulses duration is 7.76 ± 2.09 ms (range 4–18 ms), emitted at mean intervals of 4.13 ± 1.58 ms (range 1–11 ms), with a mean rate of 87.94 ± 12.54 pulses/second (range 55.56–166.67 pulses/second). The calls are upward frequency modulated, with a mean frequency modulation of 2 ± 0.65 Hz/ms (range 1.15–3.69 Hz/ms). The mean dominant frequency (coincides with the fundamental) is 3.12 ± 0.13 kHz (range 2.58–3.27 kHz). The mean minimum frequency is 2.92 ± 0.13 kHz (range 2.41–3.1 kHz), while the mean maximum frequency of 3.31 ± 0.14 kHz (range 2.84–3.53 kHz). Up to six harmonics are visible.

#### *Nymphargus* sp.

**Figure 7.**
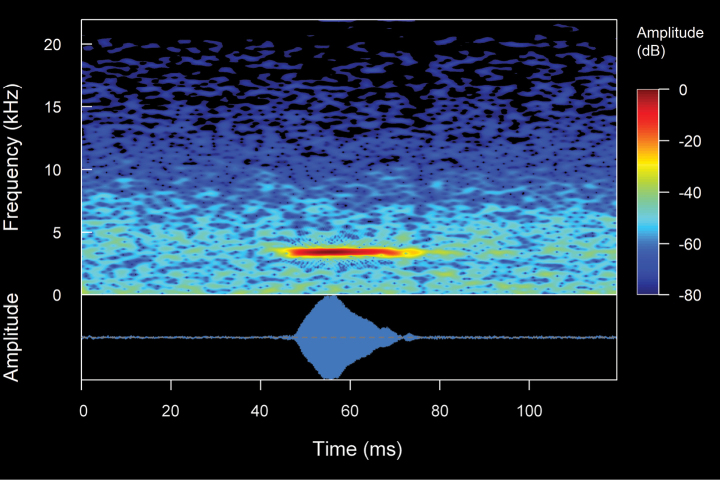
Spectrogram and oscillogram of the advertisement call of *Nymphargus* sp. (ZSFQ 6778, SVL 25. 68 mm, 10.3 °C air temperature, 96% relative humidity). Spectrogram obtained using the Hann window at 99% overlap, 256 samples of FFT size and 3 dB filter bandwidth of 248 Hz.

We recorded one male (Suppl. material 2, Table 2). The call (Fig. 7) is characterized by the emission of a non-pulsed “Tic” type note (*sensu*Duarte-Marín et al. 2022). The recorded males were calling perched on the branches of bushes in the middle of a river ~ 180 cm above the ground. The calls are loud and audible at distances of up to ca 100 m, even in the presence of a high noise level generated by the river. *Nymphargus* sp. is a nocturnal species with low to moderate vocal activity, which intensifies in the presence of light rain. The mean call duration is 23.56 ± 7.37 ms (range 15–41 ms), emitted at mean intervals of 7.94 ± 3.43 s (range 2–13.95 s) with a mean rate of 10.31 ± 7.72 calls/minute (range 4.29–29.7 calls/minute). These are upward frequency modulated calls, with a mean frequency modulation of 6.36 ± 4.6 Hz/ms (range 0–14.33 Hz/ms). The mean dominant frequency (coincides with the fundamental) is 3.41 ± 0.05 kHz (range 3.36–3.45 kHz). The mean minimum frequency is 3.27 ± 0.04 kHz (range 3.19–3.27), while the mean maximum frequency of 3.6 ± 0.04 kHz (range 3.53–3.62 kHz). Harmonics are not visible and are attributable to loud environmental background noise (recorded in the middle of a river).

### DENDROBATIDAE

#### *Hyloxalusdelatorrae* (Coloma, 1995)

**Figure 8.**
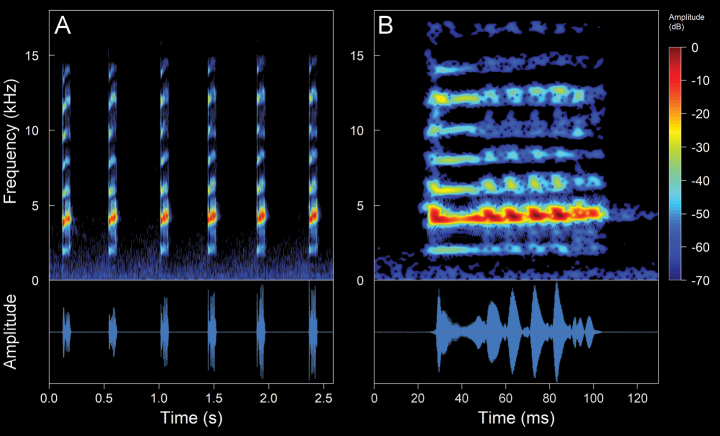
Spectrograms and oscillograms of the advertisement call of *Hyloxalusdelatorrae*. (Not collected, 17 °C air temperature, 61% relative humidity). **A** complete advertisement call **B** detail of a note with its pulses. Spectrogram obtained using the Hann window at 99% overlap, 256 samples of FFT size and 3 dB filter bandwidth of 248 Hz.

We recorded one male (Suppl. material 2, Table 2). The call (Fig. 8). The call is a continuous emission of pulsed notes that onomatopoeically resembles a “tri-tri-tri”. The recorded male was calling from a swamp densely covered with grassland. It is a diurnal species with high vocal activity, which intensifies between 11:00–12:00 h of the day. The calls are very loud and clearly audible at distances of up to ca 800 m. It is worth mentioning that a single individual was the only frog that we were able to listen to and record (not collected) in an area of ca 5 ha. The mean call duration is 2.29 ± 0.51 s (range 0.96–2.78 s), emitted at mean intervals of 3.62 ± 0.38 s (range 2.99–4.08 s) with a mean rate of 10.88 ± 2.34 calls/minute (range 9.15–16.95 calls/minute). The calls are composed of a mean of 6.1 ± 1.2 notes (range 3–7). The mean note duration is 1.46 ± 2.47 ms (range 66–77 ms), emitted at mean intervals of 363.34 ± 24.11 ms (range 324–416 ms), with a mean rate of 2.31 ± 0.13 notes/second (range 2.04–2.54 notes/second). The notes are composed of a mean of 6.72 ± 0.81 pulses (range 6–10 pulses). The mean pulses duration is 7.99 ± 5.36 ms (range 2–26 ms). The initial pulses are the longest in each note, with a mean duration of 19.88 ± 3.89 ms (range 8–26 ms). The pulses are emitted at mean intervals of 3.22 ± 1.4 ms (range 0.6–13 ms), with a mean rate of 100.04 ± 36.57 pulses/second (range 33.48–333.33 pulses/second). The calls are upward frequency modulated, with a mean frequency modulation of 2.93 ± 3.09 Hz/ms (range 0–19.28 Hz/ms). The dominant frequency is in the second harmonic, with a mean value of 4.24 ± 0.23 kHz (range 3.19–5.0 kHz), with a mean fundamental frequency of 2.12 ± 0.1 kHz (range 1.64–2.5 kHz). The mean minimum frequency is 4.04 ± 0.18 kHz (range 2.67–4.31), and the mean maximum frequency is 4.54 ± 0.2 kHz (range 3.45–5.25 kHz). Up to eight harmonics are visible, the third one having a mean frequency of 6.37 ± 0.35 kHz (range 5–7.92 kHz) and the eighth having a mean frequency of 16.66 ± 0.61 kHz (range 13.09–17.23 kHz). There might be one or two subharmonics between the first harmonic.

#### *Hyloxalus* sp.

**Figure 9.**
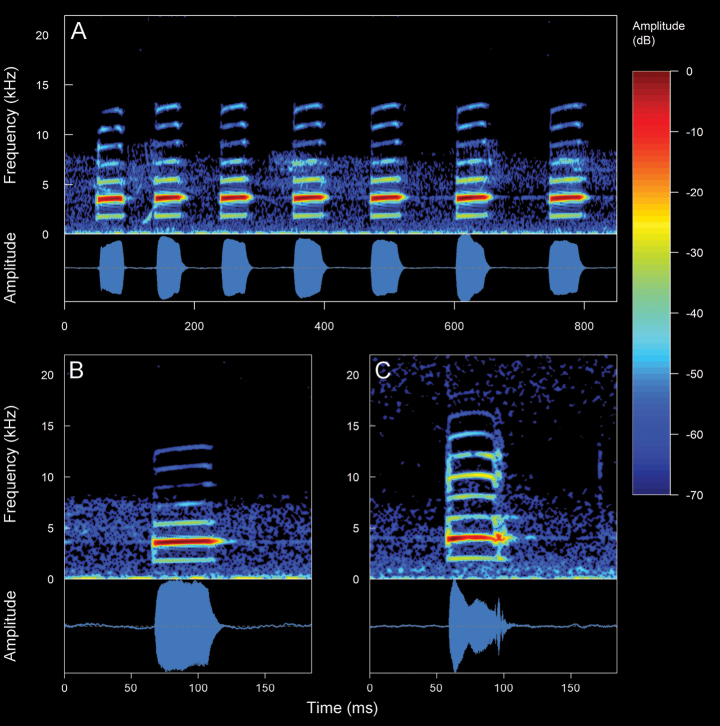
Spectrograms and oscillograms of the advertisement call of *Hyloxalus* sp. (ZSFQ 4442, SVL 15.45 mm, 16.5 °C air temperature, 66% relative humidity). **A** complete advertisement call **B** detail of a note with upward modulation **C** detail of a note with downward modulation. Spectrogram obtained using the Hann window at 99% overlap, 256 samples of FFT size and 3 dB filter bandwidth of 248 Hz.

We recorded four males (Suppl. material 2, Table 2). The call (Fig. 9). The call is a continuous emission of non-pulsed notes that onomatopoeically resembles a “ti-ti-ti”. The recorded male was calling from a swamp on the banks of a river. The area contained several springs of water from old wells that were used for human consumption. The calls are very loud and clearly audible at distances of up to ca 500 m. *Hyloxalus* sp. is a diurnal species with high vocal activity, which intensifies between 9:00–1:00 h, especially on sunny days. The mean call duration is 610.24 ± 167.58 ms (range 368–888 ms), emitted at mean intervals of 6.2 ± 5.41s (range 2.05–37.85 s) with a mean rate of 11.21 ± 4.59 calls/minute (range 1.58–24.59 calls/minute). The calls are composed of a mean of 6.27 ± 0.91 notes (range 5–8). The mean note duration is 34.66 ± 6.28 ms (range 17–51 ms), emitted at mean intervals of 75.18 ± 16.28 ms (range 48–122 ms), with a mean rate of 9.45 ± 1.76 notes/second (range 6.25–13.7 notes/second). The calls are upward frequency modulated (except for one recording that features downward modulation calls), with a mean frequency modulation of 6.22 ± 4.28 Hz/ms (range 0–29.85 Hz/ms). The dominant frequency is in the second harmonic, with a mean value of 3.91 ± 0.18 kHz (range 3.27–4.13 kHz), with a mean fundamental frequency of 1.95 ± 0.09 kHz (range 1.64–2.07 kHz). The mean minimum frequency is 3.72 ± 0.16 kHz (range 3.19–3.96 kHz), while the mean maximum frequency is 3.36 ± 0.13 kHz (range 2.9–3.63 kHz). Up to nine harmonics are visible

### HEMIPHRACTIDAE

#### *Gastrothecaorophylax* Duellman & Pyles, 1980

**Figure 10.**
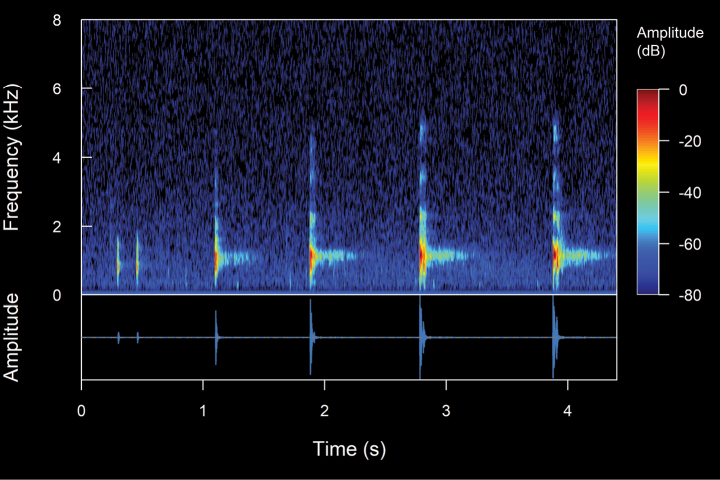
Spectrogram and oscillogram of the advertisement call of *Gastrothecaespeletia*. (DHMECN 13761, SVL 57.65 mm, 12.3 °C air temperature, 93% relative humidity). Spectrogram obtained using the Hann window at 99% overlap, 512 samples of FFT size and 3 dB filter bandwidth of 124 Hz.

We recorded four males (Suppl. material 2, Table 2). The call (Fig. 10) consists of the continuous emission of pulsed and non-pulsed notes. Their sounds are like a kind of knocking on wood that onomatopoeically resembles a “ toc, toc, troc”. The recorded males were calling perched on branches of trees and leafy shrubs ~ 2–6 m above the ground. The calls are of high intensity and clearly audible at long distances of up to ca 600 m. *Gastrothecaorophylax* is a nocturnal species with low to moderate vocal activity, which intensifies in a drizzle. The mean call duration is 3.01 ± 1.47 s (range 1.11–7.66 s), emitted at mean intervals of 18.46 ± 15.99 s (range 5.69–87.84 s), with a mean rate of 3.52 ± 1.35 calls/minute (range 0.67–6.44 calls/minute). The calls are composed of a mean of 4 ± 1.34 notes (range 1–7 notes). The mean note duration is 34.87 ± 45.98 ms (range 6–265 ms), emitted at mean intervals of 915.58 ± 256.57 ms (range 160–1421 ms), with a mean rate of 1.19 ± 0.65 notes/second (range 0.68–5.92 notes/second). The final notes consist of 1–4 pulses and are the longest in the call (range 20–219 ms). One of the distinctive characteristics of the call is the presence of two introductory notes or elements that break with the temporal uniformity of the intervals between notes. The calls are non-frequency modulated, with a mean dominant frequency (coincides with the fundamental) of 0.98 ± 0.12 kHz (range 0.78–1.21 kHz). The mean minimum frequency is 0.78 ± 0.12 kHz (range 0.52–1.12), while the mean maximum frequency is 1.16 ± 0.1 kHz (range 0.86–1.29 kHz). Up to eight harmonic partials are visible.

### STRABOMANTIDAE

#### *Niceforoniabrunnea* (Lynch, 1975)

**Figure 11.**
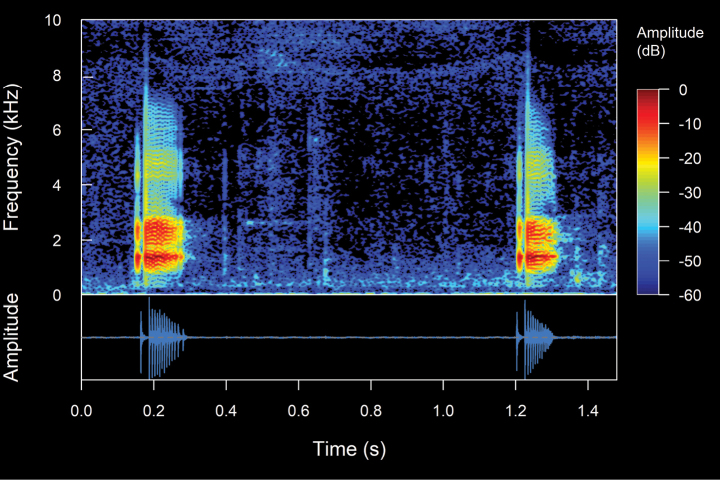
Spectrogram and oscillogram of the advertisement call of *Niceforoniabrunnea*. (ZSFQ 4507, SVL 19.8 mm, 8.2 °C air temperature, 91% relative humidity). Spectrogram obtained using the Hann window at 99% overlap, 1024 samples of FFT size and 3 dB filter bandwidth of 61.9 Hz.

We recorded two males (Suppl. material 2, Table 2). The call (Fig. 11) consists of the emission of pulsed notes. Their sounds are reminiscent of the quacking of a duck that onomatopoeically resembles a “quack, quack, quack”. The recorded males were calling from stony ground at a depth of ~ 50–150 cm below the ground surface (i.e., species of fossorial habits). The calls are of low intensity and, given the fossorial habits of the species, are unlikely to be audible even at relatively short distances. *Niceforoniabrunnea* is a crepuscular and nocturnal species with low vocal activity. The mean call duration is 1.39 ± 0.4 s (range 0.8–1.7 s), emitted at mean intervals of 18.21 ± 21.76 s (range 2.63–43.07 s), with a mean rate of 9.71 ± 10.98 calls/minute (range 1.34–22.14 calls/minute). The calls are composed of a mean of 3.2 ± 1.1 notes (range 2–4 notes). The mean note duration is 94.69 ± 11.96 ms (range 79–122 ms), emitted at mean intervals of 495.27 ± 158.54 ms (range 375–916 ms), with a mean rate of 1.77 ± 0.36 notes/second (range 0.96–2.12 notes/second). The notes are composed of a mean of 20.6 ± 2.67 pulses (range 14–24 pulses). The mean pulse duration is 2.69 ± 0.95 ms (range 1–9 ms), emitted at mean intervals of 2 ± 3.53 ms (range 0.3–21 ms), with a mean rate of 289.96 ± 91.47 pulses/second (range 41.67–500 pulses/second). In general, the intervals between pulses are almost indistinguishable. However, the introductory pulses of each note are emitted at clearly differentiated intervals (range 14–18 ms), in addition to being the longest lasting pulses (range 4–9 ms). The calls are upward frequency modulated, with a mean frequency modulation of 1.23 ± 1.25 Hz/ms (range 0–3.67 Hz/ms). The dominant frequency is between the fourth and seventh harmonic partial, with a mean value of 1.47 ± 0.11 kHz (range 1.03–1.89 kHz), while the mean fundamental frequency is 0.35 ± 0.07 kHz (range 0.22–0.56 kHz). The mean minimum frequency is 1.33 ± 0.11 kHz (range 0.95–1.72), with a mean maximum frequency of 1.59 ± 0.13 kHz (range 1.16–2.11 kHz). The harmonic series exhibits a wide frequency distribution, with a considerable number of partials visible (range 25–47 harmonics, including sidebands). In this context, the partial with the maximum frequency has a mean of 7.83 ± 0.83 kHz (range 6.91–9.72 kHz).

#### *Noblella* sp.

**Figure 12.**
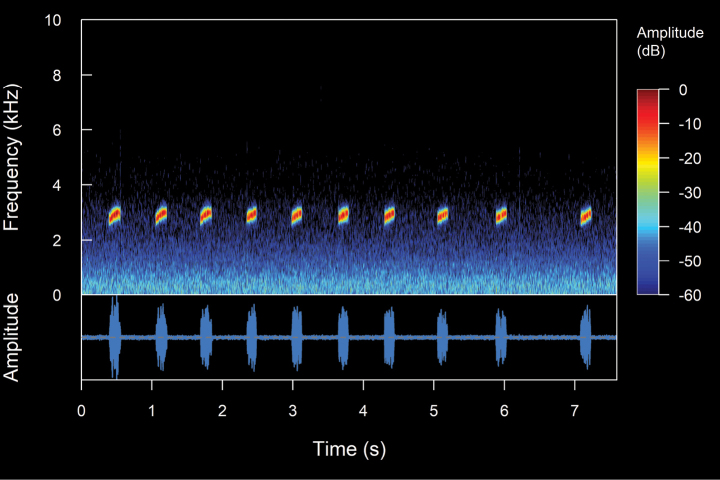
Spectrogram and oscillogram of the advertisement call of *Noblella* sp. (ZSFQ 4543, SVL 22.8 mm, 12.6 °C air temperature, 94% relative humidity). Spectrogram obtained using the Hann window at 99% overlap, 512 samples of FFT size and 3 dB filter bandwidth of 124 Hz.

We recorded 1 male (Suppl. material 2, Table 2). The call (Fig. 12) consists of the continuous emission of non-pulsed notes. Their sounds are low-intensity whistles that may be confused for the calls of a dendrobatid. The males were recorded calling from leaf litter in a mountainous area traversed by deep ravines. The calls are of moderate intensity and can be audible at distances of up to ca 50 m. *Noblella* sp. is a diurnal species with low to moderate vocal activity, which intensifies until the early afternoon (between 13:00–15:00 h). The mean call duration is 6.49 ± 0.29 s (range 6.29–6.83 s), emitted at intervals range 31.7–69.79 s, with a mean rate of 0.86–1.89 calls/minute. The calls are composed of ten notes, with a mean duration of 160.97 ± 16.42 ms (range 130–196 ms). The notes are emitted at mean intervals of 542.89 ± 131.52 ms (range 375–916 ms), with a mean rate of 1.45 ± 0.2 notes/second (range 0.84–1.77 notes/second). The calls are upward frequency modulated, with a mean frequency modulation of 2.14 ± 0.71 Hz/ms (range 0.93–3.84 Hz/ms). The mean dominant frequency (coincides with the fundamental) is 2.9 ± 0.04 kHz (range 2.84–2.93 kHz). The mean minimum frequency is 2.69 ± 0.03 kHz (range 2.67–2.76 kHz) while the mean maximum frequency is 3.1 ± 0.02 kHz (range 3.01–3.1 kHz).

#### *Pristimantisbuckleyi* (Boulenger, 1882)

**Figure 13.**
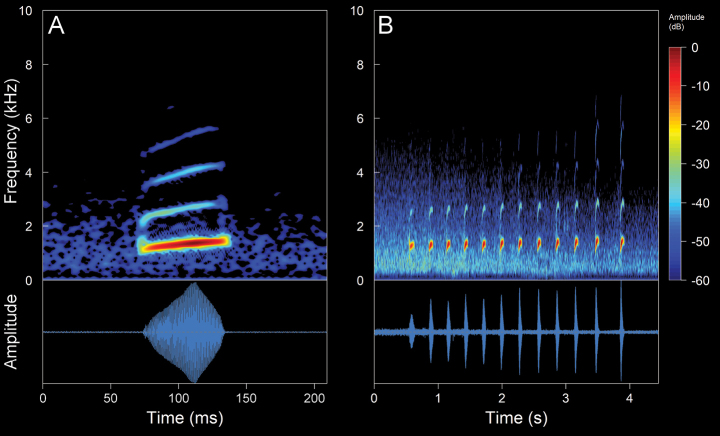
Spectrograms and oscillograms of the advertisement call of *Pristimantisbuckleyi*. **A** single call (DHMECN 13785, SVL 26.62 mm, 8.7 °C air temperature, 73% relative humidity) **B** call with continuous notes (DHMECN 13670, SVL 28.27 mm, 7.2 °C air temperature, 73% relative humidity). Spectrograms obtained using the Hann window at 99% overlap, 512 samples of FFT size and 3 dB filter bandwidth of 124 Hz.

We recorded four males (Suppl. material 2, Table 2). The call (Fig. 13) consists of the emission of a non-pulsed single note that onomatopoeically resembles a “bop”. The recorded males were calling from grasslands, swamps, and shrubby vegetation 50 to 80 cm above the ground. The calls are of low to medium intensity and can be audible at distances of up to ca 20 m. *Pristimantisbuckleyi* is a nocturnal species with low to moderate vocal activity, which intensifies until approximately 20:00 h. The mean call duration is 51.46 ± 4.77 ms (range 45–61 ms), emitted at mean intervals of 4.23 ± 0.96 s (range 1.44–5.83 s), with a mean rate of 15.12 ± 5.63 calls/minute (range 10.22–40.27 calls/minute). Normally, the call of *P.buckleyi* is composed of a single note. However, after a long call train, they emit calls that are unusually and sporadic, composed of multiple notes (in the present study, we recorded a call with 12 notes). These notes are emitted at mean intervals of 240.64 ± 34.75 ms (range 209–335 ms), with a mean rate of 3.36 ± 0.32 notes/second (range 2.56–3.64 notes/second). The calls are upward frequency modulated, with a mean frequency modulation of 1.22 ± 1.47 Hz/ms (range 0–4.78 Hz/ms). The mean dominant frequency (coincides with the fundamental) is 1.26 ± 0.07 kHz (range 1.12–1.38 kHz). The mean minimum frequency is 1.08 ± 0.07 kHz (range 1.03–1.21 kHz) while the mean maximum frequency is 1.42 ± 0.09 kHz (range 1.29–1.55 kHz). Up to seven harmonics are visible.

#### *Pristimantisfarisorum* Mueses-Cisneros, Perdomo-Castillo & Cepeda-Quilindo, 2013

**Figure 14.**
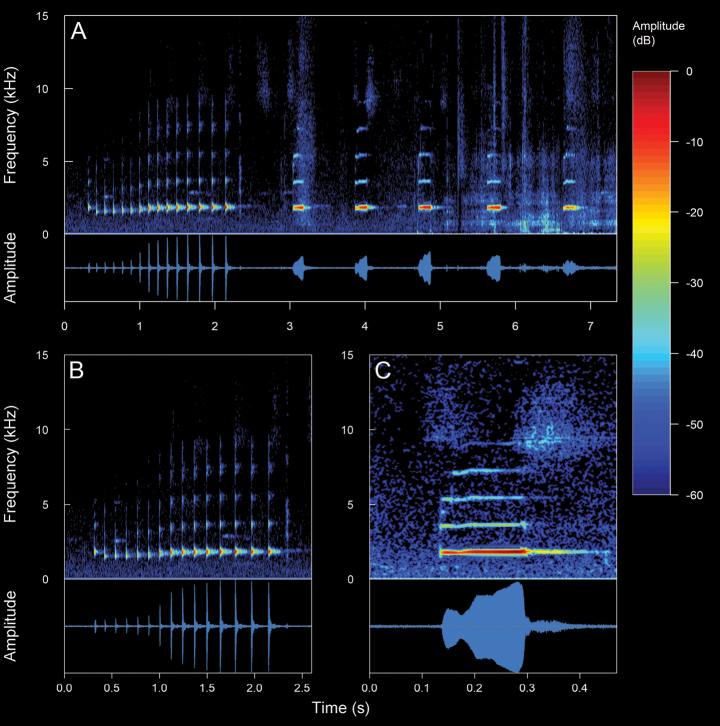
Spectrograms and oscillograms of the advertisement call of *Pristimantisfarisorum* (DHMECN 13762, SVL 32.4 mm, 10.8 °C air temperature, 98% relative humidity). **A** complete advertisement call. **B** detail of pulsed note. **C** detail of a non-pulsed note. Spectrograms obtained using the Hann window at 99% overlap, 256 samples of FFT size and 3 dB filter bandwidth of 248 Hz.

We recorded three males (Suppl. material 2, Table 2). This is a complex call (Fig. 14) consisting of the emission of a pulsed note followed by several non-pulsed notes. Their sounds are like a metallic tapping that onomatopoeically resembles a “tic”, followed by characteristic and peculiar whistling sounds that can become a high-pitched moaning. The recorded males were calling perched on branches of trees and leafy shrubs ~ 1.5–4 m above the ground. The calls are high intensity, particularly evident in the non-pulsed notes which can be audible over considerable distances. It is a crepuscular and nocturnal species with moderate vocal activity, which intensifies after a light rain or in response to the calls of nearby males. The mean call duration is 2.51 ± 1.48 s (range 1.45–6.48 s), emitted at mean intervals of 25.17 ± 5.8 s (range 17.04–33.74 s) with a mean rate of 2.27 ± 0.57 calls/minute (range 1.57–3.15 calls/minute). The calls are composed of a mean of 1.67 ± 1.61 notes (range 1–6 notes). The mean duration of the first note is 1.65 ± 0.16 s (range 1.45–1.85 s). The first note is pulsed, composed of a mean of 16.7 ± 1.64 pulses (range 15–19 pulses). The mean pulse duration is 9.16 ± 3.9 ms (range 4–24 ms), emitted at mean intervals of 109.72 ± 16.93 ms (range 64–164 ms), with a mean rate of 8.66 ± 1.35 pulses/second (range 5.59–14.49 pulses/second). The pulsed note is followed by 3–5 non-pulsed notes, which are separated by intervals of 779–875 ms. The mean non-pulsed notes duration is 173.63 ± 17.94 ms (range 141–199 ms), emitted at mean intervals of 765.5 ± 72.15 ms (range 682–848 ms), with a mean rate of 1.08 ± 0.11 notes/second (range 0.97–1.21 notes/second). The calls are non-frequency modulated. However, an upward gradual increase in frequency can be noted in the two types of notes. This upward increase is more evident in the pulsed notes, without being considered a modulated frequency (pulsed note: 0.12 ± 0.56 Hz/ms; non-pulsed note 0.91 ± 0.35 Hz/ms; *sensu*Emmrich et al. 2020). In certain calls, the frequency of the first pulse is usually higher and breaks the upward hegemony of the frequency of the other elements of the call. The mean dominant frequency (coincides with the fundamental) is 1.64 ± 0.15 kHz (range 1.38–1.89 kHz). The mean minimum frequency is 1.48 ± 0.15 kHz (range 1.21–1.72 kHz) with a mean maximum frequency of 1.83 ± 0.14 kHz (range 1.55–2.07 kHz). Up to eight harmonics are visible.

#### *Pristimantisfestae* (Peracca, 1904)

**Figure 15.**
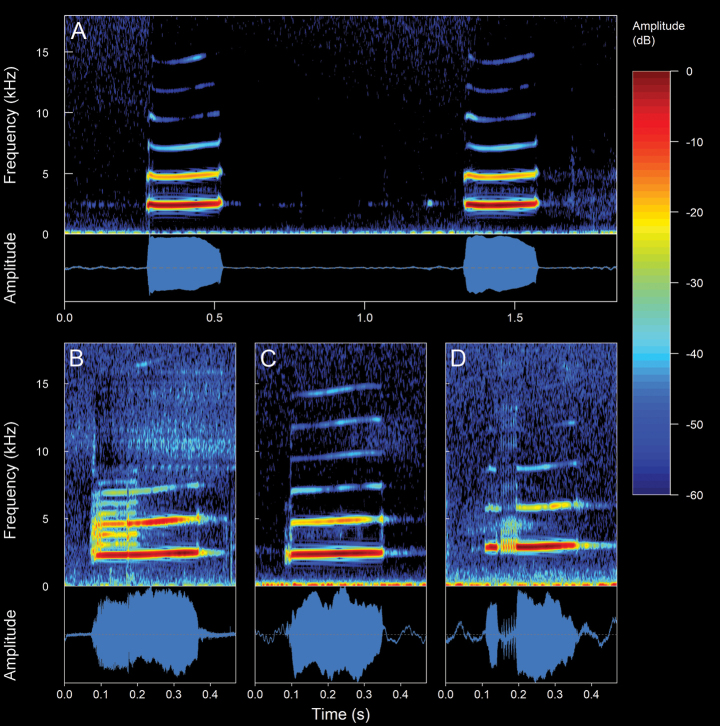
Spectrograms and oscillograms of the advertisement call of *Pristimantisfestae*. **A** detail of a complete advertisement call with two notes (ZSFQ 4440, SVL 18.7 mm, 7.7 °C air temperature, 85% relative humidity) **B** call with visible side bands (ZSFQ 4525, SVL 15.73 mm, 8.1 °C air temperature, 93% relative humidity) **C** single call with its characteristic harmonics (ZSFQ 4516, SVL 16.75 mm, 10.2 °C air temperature, 76% relative humidity) **D** detail of pulsed note (ZSFQ 4528, SVL 13.25 mm, 11.6 °C air temperature, 82% relative humidity). Spectrograms obtained using the Hann window at 99% overlap, 512 samples of FFT size and 3 dB filter bandwidth of 124 Hz.

We recorded eight males (Suppl. material 2, Table 2). The call (Fig. 15) consists of the emission of non-pulsed and pulsed notes (mostly non-pulsed). The calls are melodious whistles, emitted from inside hollow logs, leaf litter, pajonal (a group of tall herbaceous plants of the genera *Calamagrostis* and *Agrostis*), and ferns. The calls are of medium to high intensity, with sounds that can be audible at distances of up to ca 50 m. *Pristimantisfestae* is a diurnal and nocturnal species, with low to moderate vocal activity, which intensifies in the presence of light rain. The mean call duration is 0.98 ± 0.71 s (range 0.15–3.2 s), emitted at mean intervals of 8.66 ± 2.52 s (range 4.95–19.37 s) with a mean rate of 6.73 ± 1.85 calls/minute (range 3.06–11.7 calls/minute). The calls are composed of a mean of 1.72 ± 0.58 notes (range 1–3 notes). The mean note duration is 249.29 ± 29.61 ms (range 191–320 ms), emitted at mean intervals of 782.96 ± 215.01 ms (range 480–1497 ms), with a mean rate of 1 ± 0.18 notes/second (range 0.58–1.41 notes/second). Some notes have the peculiarity of being composed of a mean of 3.47 ± 2.75 pulses (range 1–10 pulses). The mean pulse duration is 12.34 ± 10.7 ms (range 2–55 ms), emitted at mean intervals of 5.98 ± 4.51 ms (range 1–21 ms), with a mean rate of 94.5 ± 64.8 pulses/second (range 16.13–250 pulses/second). The calls are upward frequency modulated, with a mean frequency modulation of 1.05 ± 0.49 Hz/ms (range 0–3.05 Hz/ms). The mean dominant frequency (coincides with the fundamental) is 2.58 ± 0.22 kHz (range 2.33–3.01 kHz). The mean minimum frequency is 2.41 ± 0.21 kHz (range 2.15–2.76 kHz), while the mean maximum frequency is 2.75 ± 0.2 kHz (range 2.5–3.1 kHz). Up to eight harmonics are discernible, with two to four subharmonics evident between each harmonic. These subharmonics are observable in certain calls and specific individuals, and do not occur throughout the entire call.

#### *Pristimantishuicundo* (Guayasamin, Almeida-Reinoso & Nogales-Sornosa, 2004)

**Figure 16.**
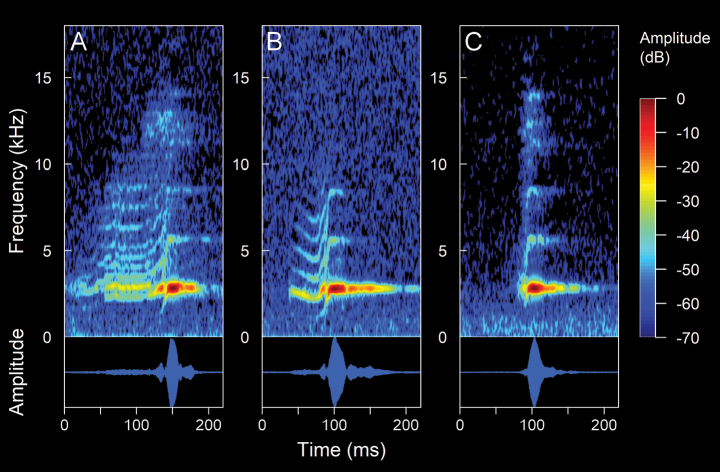
Spectrograms and oscillograms of the different types of advertisement calls present in *Pristimantishuicundo*. **A** inharmonic call (Not collected, 10 °C air temperature, 61% relative humidity) **B** downward call (DHMECN 13777, SVL 17.7 mm, 9.8 °C air temperature, 96% relative humidity) **C** single call (DHMECN 13791, SVL 22.4 mm, 9.7 °C air temperature, 77% relative humidity). Spectrograms obtained using the Hann window at 99% overlap, 256 samples of FFT size and 3 dB filter bandwidth of 248 Hz.

We recorded five males (Suppl. material 2, Table 2). The call (Fig. 16) consists of the emission of a non-pulsed note. Their sounds are like a kind of a slight snoring mixed with a metallic tapping that onomatopoeically resembles a “tuic”. The recorded males were calling from inside bromeliads (commonly referred to as huicundos) or perching on branches of trees, ~ 1–5 m above the ground. The calls are of medium intensity and can be audible over long distances due to the arboreal habits. *Pristimantishuicundo* is a nocturnal species with low vocal activity. The mean call duration is 76.06 ± 26.04 ms (range 41–175 ms), emitted at mean intervals of 5.82 ± 1.97 s (range 2.87–9.32 s) with a mean rate of 11.49 ± 4.24 calls/minute (range 6.36–20.27 calls/minute). The calls are frequency modulated between upward and downward (mostly upward), with a mean frequency modulation of 2.65 ± 1.86 Hz/ms (range 0–6.29 Hz/ms). The mean dominant frequency (coincides with the fundamental) is 2.8 ± 0.07 kHz (range 2.76–2.93 kHz). The mean minimum frequency is 2.63 ± 0.07 kHz (range 2.58–2.76 kHz), with a mean maximum frequency of 2.98 ± 0.07 kHz (range 2.93–3.1 kHz). Up to five harmonics are visible, with one sideband between each harmonic. The second harmonic partial has a mean frequency of 5.59 ± 0.14 kHz (range 5.51–5.86 kHz) and the fifth a mean frequency of 13.92 ± 0.28 kHz (range 13.78–14.64 kHz). In addition, two elements are observed in the call of *P.huicundo*. An introductory element of low intensity, almost imperceptible, which generates a sound similar to a slight snoring. A final element of high intensity, which generates a sound similar to a metallic tapping. These elements, particularly the introductory one, indicate a variability in the spectral and temporal characteristics. From these variations, three different types of calls can be identified: inharmonic calls. These types of calls are characterized by upward frequency modulation, with sidebands present between each harmonic. ***Downward calls***. These types of calls are characterized by downward frequency modulation, upward in the final element of the call. There are several visible harmonics, without the presence of sidebands. ***Single calls***. They are characterized by the absence of an introductory element and the presence of several harmonics. It is important to note that the distinctive feature of the *P.huicundo* call is the difference in the sequence of the harmonic series between the introductory and final element. This marks a tonal difference in the same emission.

#### *Pristimantisocreatus* (Lynch, 1981)

**Figure 17.**
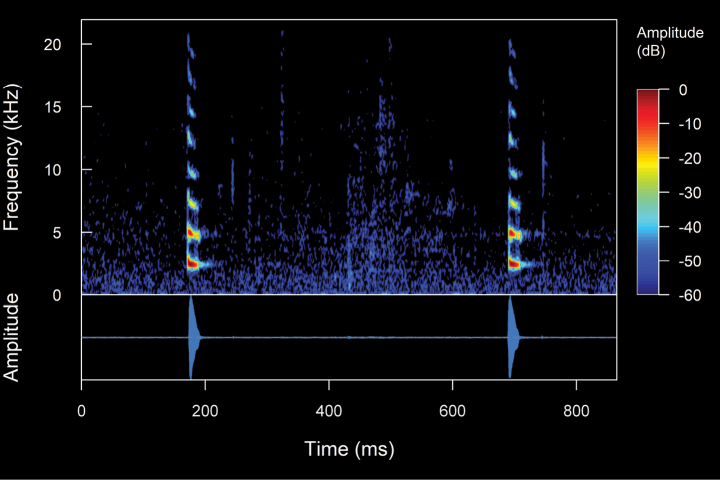
Spectrogram and oscillogram of the advertisement call of *Pristimantisocreatus*. (DHMECN 13654, SVL 13.35 mm, 8.2 °C air temperature, 88% relative humidity). Spectrogram obtained using the Hann window at 99% overlap, 256 samples of FFT size and 3 dB filter bandwidth of 248 Hz.

We recorded six males (Suppl. material 2, Table 2). The call (Fig. 17) consists of the emission of a non-pulsed note. Their sounds are like a kind of metallic tapping that onomatopoeically resembles a “tic”. The recorded males were calling from within the inside adventitious tree roots, leaf litter, pajonal (a group of tall herbaceous plants of the genera *Calamagrostis* and *Agrostis*), and from inside spiny bromeliads of the genus *Puya* (commonly referred to as achupallas). The calls are of low to medium intensity and can be audible at distances of up to ca 10 m. *Pristimantisocreatus* is a diurnal and nocturnal species with low to moderate vocal activity, which intensifies in the middle or after rain. The mean call duration is 18.12 ± 2.49 ms (range 15–23 ms), emitted at mean intervals of 528.62 ± 163.8 ms (range 393–916 ms), with a mean rate of 1.95 ± 0.44 calls/minute (range 1.07–2.44 calls/minute). The calls are downward frequency modulated, with a mean frequency modulation of 14.15 ± 4.45 Hz/ms (range 3.78–23.89 Hz/ms). The mean dominant frequency (coincides with the fundamental) is 2.4 ± 0.17 kHz (range 2.15–2.76 kHz). The mean minimum frequency is 2.2 ± 0.17 kHz (range 1.98–2.5 kHz), while the mean maximum frequency is 2.58 ± 0.18 kHz (range 2.33–2.84 kHz). Up to eight harmonics are visible.

#### *Pristimantissupernatis* (Lynch, 1979)

**Figure 18.**
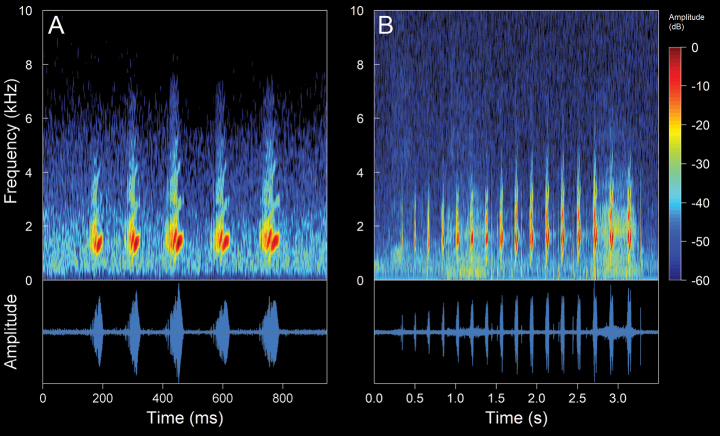
Spectrograms and oscillograms of the advertisement call of *Pristimantissupernatis*. **A** advertisement call, common and characteristic (DHMECN 13658, SVL 27.25 mm, 6.7 °C air temperature, 83% relative humidity) **B** unusual, explosive and sporadic advertisement call (DHMECN 13820, SVL 25.65 mm, 10 °C air temperature, 79% relative humidity). Spectrograms obtained using the Hann window at 99% overlap, 256 samples of FFT size and 3 dB filter bandwidth of 248 Hz.

We recorded two males (Suppl. material 2, Table 2). The call (Fig. 18) consists of the continuous emission of non-pulsed notes. Their sounds are like a kind of throaty chuckle, reminiscent of the cackling of a bird. The recorded males were calling perched on trees and shrub branches, ~ 0.8–3 m above the ground. The calls are of medium intensity and clearly audible, even over considerable distances. *Pristimantissupernatis* is a nocturnal species with low vocal activity, which emits calls fortuitously and without following a constant pattern. The mean call duration is 1359.35 ± 1273.35 ms (range 621–2830 ms), emitted at intervals of 3.92 s, with a rate of 13.19 calls/minute. The calls are composed of a mean of 8.67 ± 6.35 notes (range 5–16 notes), with a mean note duration of 44.54 ± 12.56 ms (range 22–66 ms). It should be noted that the call composed of 16 notes, is a sporadic and untimely call that usually follows a call train of short calls (composed of ≤ 5 notes). The mean intervals between notes are 127.13 ± 30.57 ms (range 74–167 ms), with a mean rate of 6.06 ± 1.12 notes/second (range 4.61–8.62 notes/second). The notes are composed of a mean of 3.32 ± 1.04 pulses (range 2–5 pulses). The mean pulse duration is 10.96 ± 8.13 ms (range 2–28 ms), emitted at mean intervals of 2.38 ± 1.71 ms (range 1–10 ms), with a mean rate of 146.14 ± 83.52 pulses/second (range 45.45–333.33 pulses/second). The dominant frequency is in the second harmonic partial, with a mean value of 1.43 ± 0.08 kHz (range 1.29–1.55 kHz). The mean fundamental frequency is 0.71 ± 0.04 kHz (range 0.6–0.78 kHz), with up to 15 harmonic partials visible. The mean minimum frequency is 1.21 ± 0.08 kHz (range 1.03–1.38 kHz), while the mean maximum frequency is 1.71 ± 0.1 kHz (range 1.46–1.98 kHz). This is a complex call at both the temporal and spectral levels. It has different tonalities and intensities throughout its emission. From this perspective, the call of *P.supernatis* call presents different elements (clarifying that they are not notes or pulses). The introductory element, composed of pulses of short duration, with harmonic partials that are not very distinguishable. The middle element, which has an upward frequency modulation, numerous harmonic partials, and visible sidebands. The final element, which has an upward frequency modulation, lacks visible sidebands and harmonics with different frequency values. This indicates that the values of the dominant frequency and harmonics differ from those of the other elements, which results in a distinct tonality within the same emission. Furthermore, the unique tonality differentiation observed in the vocalizations of *Pristimantissupernatis* can be described as a call with biphonation segments, augmenting both temporal and spectral variability. This phenomenon has been documented in other species (e.g., Brito et al. 2017; Zhang et al. 2017; Hepp et al. 2019).

#### *Pristimantisthymelensis* (Lynch, 1972)

**Figure 19.**
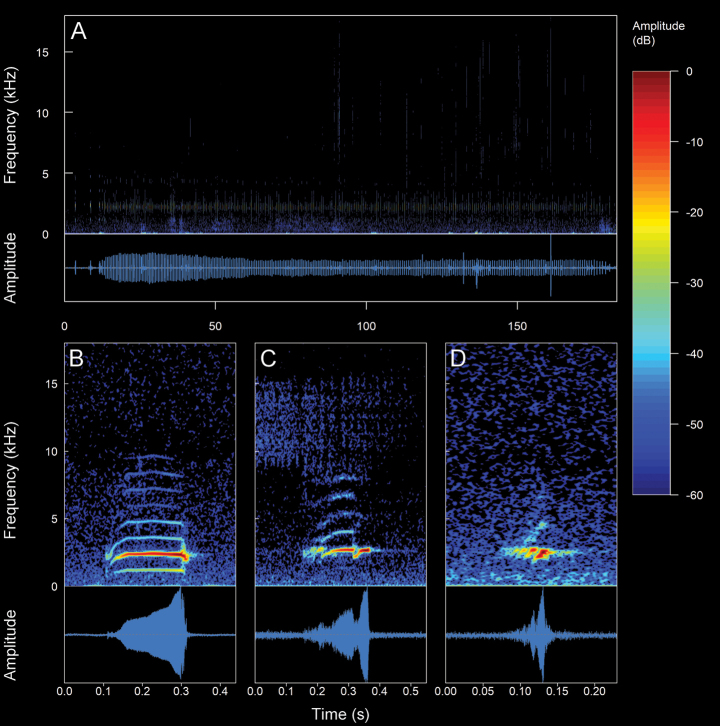
Spectrograms and oscillograms of the different types of advertisement calls present in *Pristimantisthymelensis*. **A** complete call train of 84 calls **B** single calls (**A, B** not collected individual) **C** complex calls **D** pulsed calls (C, D DHMECN 13786, SVL 19.98 mm, 4 °C air temperature, 90% relative humidity). Spectrograms obtained using the Hann window at 99% overlap, 512 samples of FFT size and 3 dB filter bandwidth of 124 Hz.

We recorded two males (Suppl. material 2, Table 2). The call (Fig. 19) consists of the emission of non-pulsed and pulsed notes. Their sounds are very varied and usually resemble a hoarse squeal or moan range low- to high-pitched. The recorded males were calling from within inside the tall grasses of the genus *Cortaderia*, ferns, bromeliads of the genus *Puya* (achupallas), and perched on branches of shrubs and among the leaves of frailejones of the genus *Espeletia* ~ 20–60 cm from above the ground. The calls are of medium to high intensity and can be audible over long distances. *Pristimantisthymelensis* is a diurnal, crepuscular, and nocturnal species, with low vocal activity. It is important to note that calls are emitted in a solitary and sporadic manner, except when between 84 and 386 consecutive calls are emitted within a period of between two and three minutes. In such cases, continuous emissions would be regarded as a complete call train. The calls of *P.thymelensis* show variability and differences in their spectral and temporal values, which makes it difficult to generalize the call of this species under a single pattern. However, considering these differences, *P.thymelensis* has three distinct different call types. ***Complex calls***. The mean duration of this type is 158.8 ± 41.73 ms (range 93–204 ms), emitted at mean intervals of 7.38 ± 9.7 s (range 1.24–24.53 s), with a mean rate of 19.5 ± 15.98 calls/minute (range 2.43–45.11 calls/minute). The calls are composed of a mean of 2.83 ± 0.41 pulses (range 2–3 pulses), with a mean pulse duration of 14.04 ± 14.59 ms (range 3–114 ms). The dominant frequency is in the second harmonic partial, with a mean value of 2.63 ± 0.09 kHz (range 2.5–2.76 kHz). They are upward frequency modulated, with a mean fundamental frequency of 1.38 kHz. The mean minimum frequency is 2.37 ± 0.11 kHz (range 2.15–2.5 kHz), while the mean maximum frequency is 2.8 ± 0.09 kHz (range 2.58–2.93 kHz). The complex call is divided into an introductory, middle, and final element. Each of these elements has different spectral characteristics. In this context, this type of call has up to six harmonics, which are only visible in the middle element. It should be noted that this type of call marks the beginning of the call train. ***Single calls***. They are unusual, uncommon calls, which have no division of elements and are composed of a single note. The mean duration of this type is146.25 ± 55.42 ms (range 64–185 ms), emitted at mean intervals of 2.27 ± 1.99 s (range 0.62–4.84 s) with a mean rate of 45.82 ± 35.89 calls/minute (range 11.98–86.96 calls/minute). The dominant frequency is in the second harmonic, with a value of 2.41 kHz. The calls are downward and upward frequency modulated, with a fundamental frequency of 1.21 kHz. The mean minimum frequency is 2.08 ± 0.07 kHz (range 1.98–2.15 kHz), while the maximum frequency is 2.5 kHz. Up to eight harmonics are visible, the third having a frequency of 3.62 kHz and the eighth a frequency of 9.65 kHz. They are unusual, uncommon calls, which have no division of elements and are composed of a single note. ***Pulsed calls***. The mean call duration is 47.79 ± 9.36 ms (range 31–67 ms), emitted at intervals of 0.57 ± 0.13 s (range 0.41–0.99 s), with a rate of 99.65 ± 18.99 calls/minute (range 58.25–130.43 calls/minute). The calls are composed of a mean of 3.53 ± 1.3 pulses (range 2–6 pulses). The mean pulse duration is 10.67 ± 5.07 ms (range 3–27 ms), the last pulses being the longest of the call, with a mean duration of 16.57 ± 5.11 ms (range 11–27 ms). Calls downward frequency modulated, with a gradual and continuous decrease in frequency over the course of the call. The dominant frequency is in the second harmonic, with a mean value of 2.63 ± 0.09 kHz (range 2.24–2.76 kHz), while the mean fundamental frequency is 1.2 ± 0.02 kHz (range 1.12–1.21 kHz). The mean minimum frequency is 2.26 ± 0.1 kHz (range 2.07–2.5 kHz), with a mean maximum frequency of 2.77 ± 0.12 kHz (range 2.58–2.84 kHz). Up to six harmonics are visible, The pulsed call is not emitted as an isolated call (i.e., without being part of a call train). This type represents the most intense part of the call train, with intervals between calls becoming shorter as the call train progresses.

#### *Pristimantisunistrigatus* (Günther, 1859)

**Figure 20.**
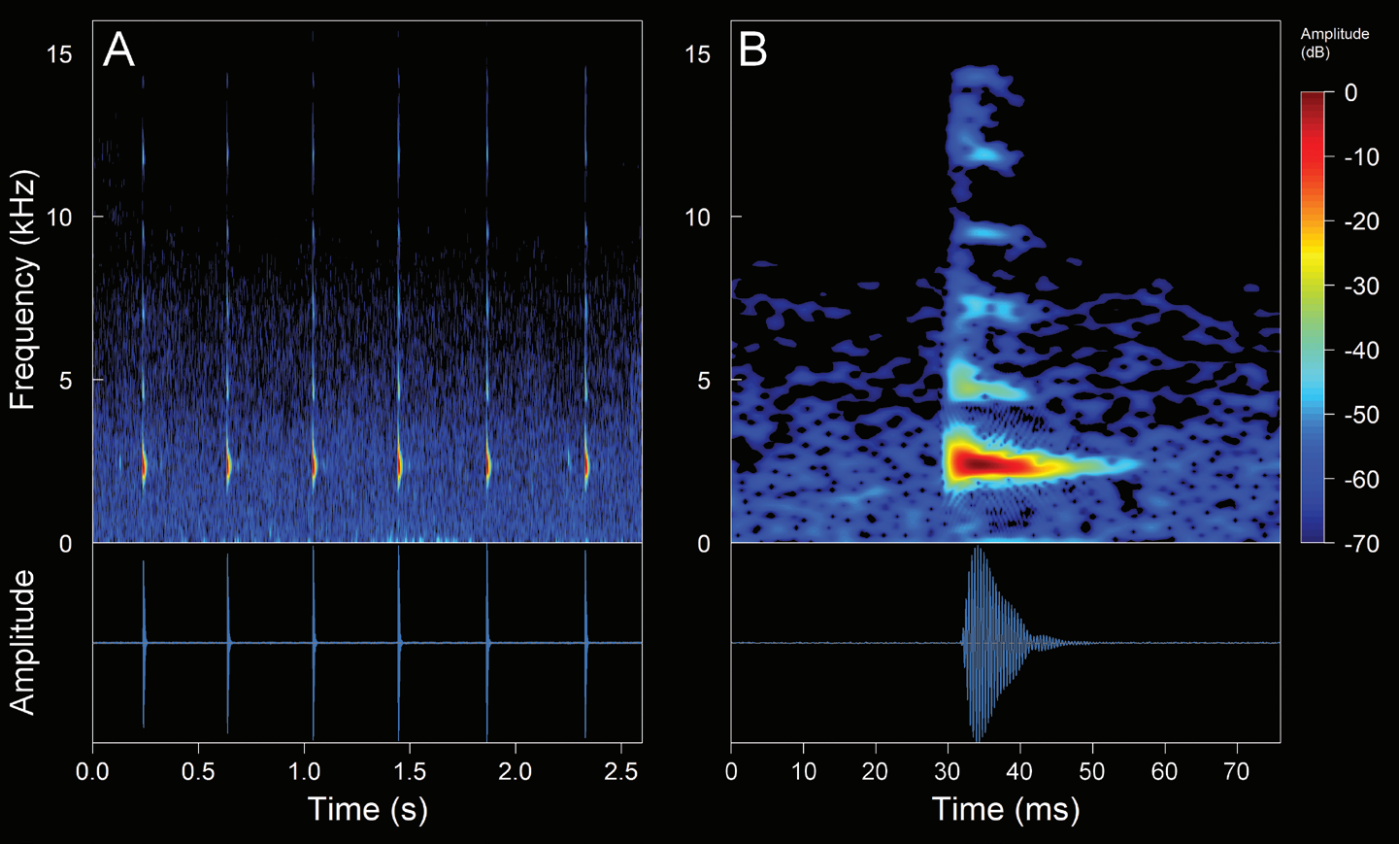
Spectrograms and oscillograms of the advertisement call of *Pristimantisunistrigatus* (ZSFQ 4549, SVL 20.25 mm, 9.8 °C air temperature, 62% relative humidity). **A** complete advertisement call **B** detail of a note. Spectrogram obtained using the Hann window at 99% overlap, 256 samples of FFT size and 3 dB filter bandwidth of 248 Hz.

We recorded four males (Suppl. material 2, Table 2). The call (Fig. 20) consists of the continuous emission of non-pulsed notes. Their sounds are like a kind of metallic tapping that onomatopoeically resembles a “tic-tic-tic”. The recorded males were calling from grasslands, leaf litter and in cultivated areas (this species does not inhabit forests), or perching on shrubby vegetation ~ 20–60 cm above the ground. The calls are of medium to high intensities and may be audible over long distances, particularly in open fields. *Pristimantisunistrigatus* is a diurnal, crepuscular, and nocturnal species, with moderate to high vocal activity, which intensifies during or after a drizzle. The mean call duration is 1.54 ± 0.47 ms (range 0.67–2.13 ms), emitted at mean intervals of 9.18 ± 3.49 s (range 4.87–16.27 s) with a mean rate of 6.31 ± 2.16 calls/minute (range 3.35–10.6 calls/minute). The calls are composed of a mean of 5.48 ± 1.57 notes (range 3–8 notes). The mean note duration is 7.47 ± 2.7 ms (range 4–15 ms), emitted at mean intervals of 343.48 ± 53.58 ms (range 250–459 ms), with a mean rate of 2.92 ± 0.47 notes/second (range 2.13–3.91 notes/second). The calls are non-frequency modulated, with downward frequency modulated notes. The mean dominant frequency (coincides with the fundamental) is 2.51 ± 0.2 kHz (range 1.98–2.93 kHz). The mean minimum frequency is 2.26 ± 0.19 kHz (range 1.81–2.15 kHz), while the mean maximum frequency is 2.86 ± 0.27 kHz (range 2.33–3.36 kHz). Up to six harmonics are visible.

#### *Pristimantis* sp. (*Pristimantisridens* species group)

**Figure 21.**
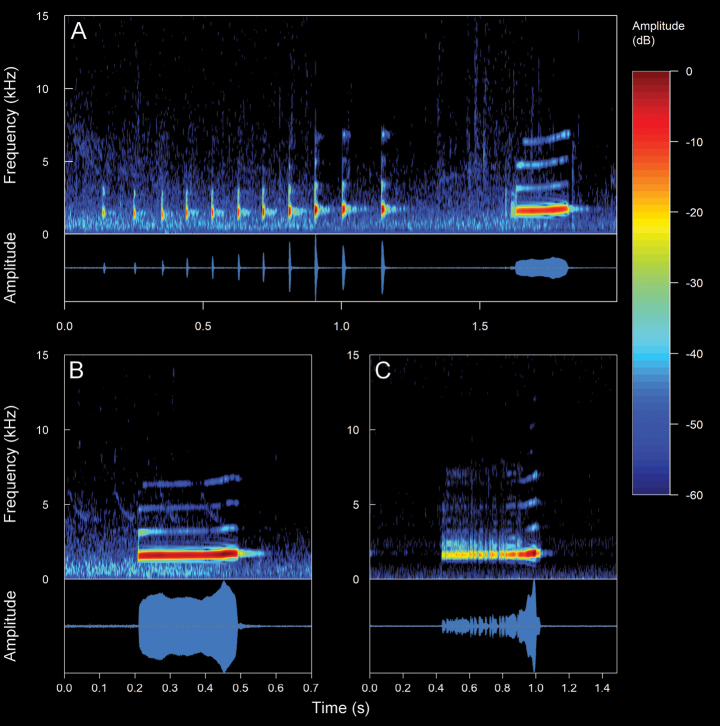
Spectrograms and oscillograms of the advertisement call of *Pristimantis* sp. 1 *ridens* group **A** Complete advertisement call **B** detail of a non-pulsed note (**A, B**DHMECN 13752, SVL 34.4 mm, 11 °C air temperature, 85% relative humidity) **C** detail of a pulsed secondary note (**A, B**ZSFQ 4499, SVL 39.1 mm, 11.6 °C air temperature, 90% relative humidity). Spectrograms obtained using the Hann window at 99% overlap, 256 samples of FFT size and 3 dB filter bandwidth of 248 Hz.

We recorded four males (Suppl. material 2, Table 2). This is a complex call (Fig. 21) consisting of a pulsed note followed by several non-pulsed notes. The call of this species is very similar to that of *P.farisorum* in both structure and sound (these 2 species are cryptic). That is, metallic tapping that onomatopoeically resembles a “tic”, followed by characteristic and peculiar whistling sounds that can become a high-pitched moaning. The recorded males were calling perched on branches of trees and leafy shrubs ~ 1–4 m above the ground. The calls are high intensity, particularly evident in the non-pulsed notes which can be audible over long distances. This is a crepuscular and nocturnal species with moderate vocal activity, which intensifies after a light rain or in response to the calls of nearby males. The mean call duration is 1.91 ± 0.68 s (range 0.6–3.35 s), emitted at mean intervals of 11.81 ± 4.07 s (range 3.31–22.66 s), with a mean rate of 4.79 ± 2.05 calls/minute (range 2.39–12.3 calls/minute). The calls are composed of a mean of 1.84 ± 0.69 notes (range 1–4 notes). The mean duration of the first note is 1241.94 ± 306.06 s (range 679–1603 s). The first note is pulsed, composed of a mean of 11.68 ± 2.24 pulses (range 7–15 pulses). The mean pulse duration is 8.33 ± 7.57 ms (range 3–50 ms), emitted at mean intervals of 91.11 ± 10.4 ms (range 82–140 ms), with a mean rate of 10.3 ± 1.02 pulses/second (range 6.33–11.36 pulses/second). The pulsed note is followed by 1–4 non-pulsed notes, which are separated by intervals of 414–960 ms. The mean non-pulsed notes duration is 338.91 ± 131.04 ms (range 141–199 ms), emitted at mean intervals of 631.2 ± 137.61 ms (range 414–960 ms), with a mean rate of 0.69 ± 0.23 notes/second (range 0.39–1.14 notes/second). Some secondary notes (i.e., those that followed the first note) are composed of a mean of 11.5 ± 4.95 pulses (range 8–15 pulses). Secondary note pulses have a mean duration of 41.72 ± 49.12 ms (range 9–184 ms), emitted at mean intervals of 8.24 ± 4.07 ms (range 2–17 ms), with a mean rate of 37.11 ± 19.6 pulses/second (range 2.76–71.43 pulses/second). The calls are non-frequency modulated. However, an upward gradual increase in frequency can be noted in the two types of notes. This upward increase is more evident in the pulsed notes but is not considered a modulated frequency (pulsed note: 0.22 ± 0.17 Hz/ms; secondary notes 0.62 ± 0.58 Hz/ms; *sensu*Emmrich et al. 2020). The mean dominant frequency (coincides with the fundamental) is 1.64 ± 0.11 kHz (range 1.38–1.81 kHz), The mean minimum frequency is 1.44 ± 0.12 kHz (range 1.03–1.64 kHz), while the mean maximum frequency is 1.85 ± 0.1 kHz (range 1.55–1.98 kHz). Up to seven harmonics are visible.

#### *Pristimantis* sp. 2 (*Pristimantismyersi* species group)

**Figure 22.**
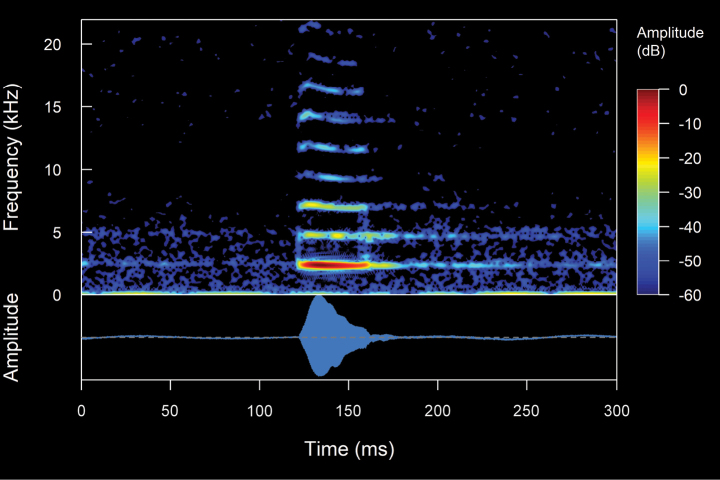
Spectrogram and oscillogram of the advertisement call of *Pristimantis* sp. 2 *myersi* group (ZSFQ 4565, SVL 18.86 mm, 11.5 °C air temperature, 89% relative humidity). Spectrogram obtained using the Hann window at 99% overlap, 256 samples of FFT size and 3 dB filter bandwidth of 248 Hz.

We recorded 12 males (Suppl. material 2, Table 2). The call (Fig. 22) consists of the emission of a non-pulsed note. Their sounds are like a kind of metallic tapping that onomatopoeically resembles a “tic”. The recorded males were calling from dense undergrowth with abundant bryophytes, leaf litter, within hollow trunks, and the base and interior of adventitious tree roots. The calls are of medium to high intensities and they can be audible at distances of up to ca 150 m. This species is active during the day and night, with moderate to high vocal activity, which intensifies during or after rain and in response to the calls of nearby males. The mean call duration is 225.79 ± 263.9 ms (range 8–1042 ms), emitted at mean intervals of 3.8 ± 1.71 s (range 2.15–21.45 s), with a mean rate of 16.23 ± 3.96 calls/minute (range 2.79–27.03 calls/minute). The calls are composed of a mean of 1.6 ± 0.63 notes (range 1–3 notes). The mean note duration is 14.01 ± 4.89 ms (range 9–39 ms), emitted at mean intervals of 407.51 ± 91.52 ms (range 302–798 ms), with a mean rate of 2.46 ± 0.41 notes/second (range 1.24–3.18 notes/second). The calls are downward frequency modulated (upward in some calls), with a mean frequency modulation of 6.23 ± 6.4 Hz/ms (range 0–34.4 Hz/ms). The mean dominant frequency (coincides with the fundamental) is 2.63 ± 0.13 kHz (range 2.33–2.84 kHz). The mean minimum frequency is 2.42 ± 0.12 kHz (range 2.15–2.67 kHz), while the mean maximum frequency is 2.78 ± 0.13 kHz (range 2.5–3.01 kHz). Up to eight harmonics are visible.

#### *Pristimantis* sp. 3 (*Pristimantismyersi* species group)

**Figure 23.**
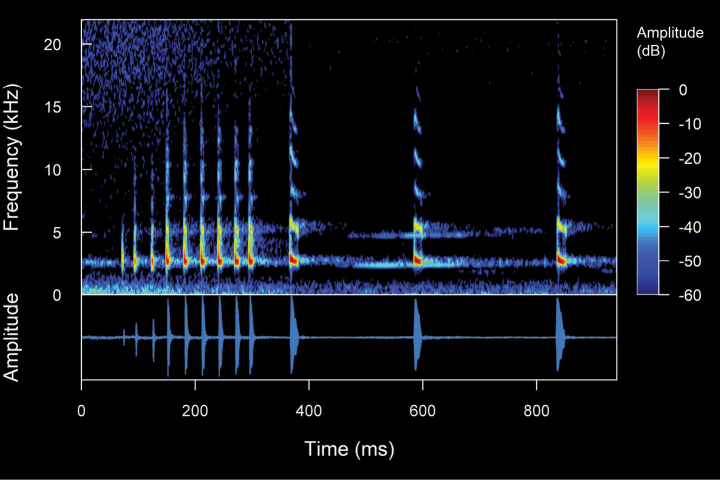
Spectrogram and oscillogram of the advertisement call of *Pristimantis* sp. 3 *myersi* group (ZSFQ 4512, SVL 13.35 mm, 9.9 °C air temperature, 76% relative humidity). Spectrogram obtained using the Hann window at 99% overlap, 256 samples of FFT size and 3 dB filter bandwidth of 248 Hz.

We recorded nine males (Suppl. material 2, Table 2). This is a complex call (Fig. 23) consisting of a pulsed note seconded by several non-pulsed notes. Their sounds are like a constant metallic tapping that onomatopoeically resembles a “tic”. The recorded males were calling from leaf litter, inside hollow trunks, the base and interior of trees, frailejones of the genus *Espeletia*, and ferns. The calls are of low to high intensity, with sounds that can be audible at distances of up to ca 30 m. This is a crepuscular and nocturnal species (with little diurnal activity), with moderate to high vocal activity, which intensifies after a rain. The mean call duration is 630.17 ± 281.68 ms (range 207–1294 ms), emitted at mean intervals of 3.89 ± 1.2 s (range 2.29–12.92 s), with a mean rate of 14.21 ± 3.28 calls/minute (range 7.03–20.62 calls/minute). The calls are composed of a mean of 3.01 ± 1.11 notes (range 1–5 notes). The mean duration of the first note is 239.18 ± 79.5 ms (range 104–490 s). The first note is pulsed, composed of a mean of 6.5 ± 1.88 pulses (range 2–12 pulses), The mean pulse duration is 6.08 ± 2.41 ms (range 1–19 ms), emitted at mean intervals of 39.33 ± 14.21 ms (range 4–93 ms), with a mean rate of 24.58 ± 8.68 pulses/second (range 10.2–111.11 pulses/second). The first note is followed by 1–4 non-pulsed notes, which are separated by a mean interval of 95.88 ± 30.08 (range 53–258 ms). The mean non-pulsed notes duration is 13.02 ± 3.71 ms (range 6–29 ms), emitted at mean intervals of 254.13 ± 44.82 ms (range 191–644 ms), with a mean rate of 3.54 ± 0.8 notes/second (range 1.34–9.62 notes/second. The calls are non-frequency modulated, exhibiting downward modulation in their notes (Pulsed note: 1.31 ± 0.77 Hz/ms; non-pulsed notes 22.42 ± 8.07 Hz/ms). The mean dominant frequency (coincides with the fundamental) is 2.55 ± 0.09 kHz (range 2.33–2.84 kHz). The mean minimum frequency is 2.34 ± 0.09 kHz (range 2.07–3.19 kHz), while the mean maximum frequency is 2.85 ± 0.18 kHz (range 2.58–3.79 kHz). Up to eight harmonics are visible. In the pulsed note, the presence of one or two sidebands between harmonics is observed.

#### *Pristimantis* sp. 4 (*Pristimantismyersi* species group)

**Figure 24.**
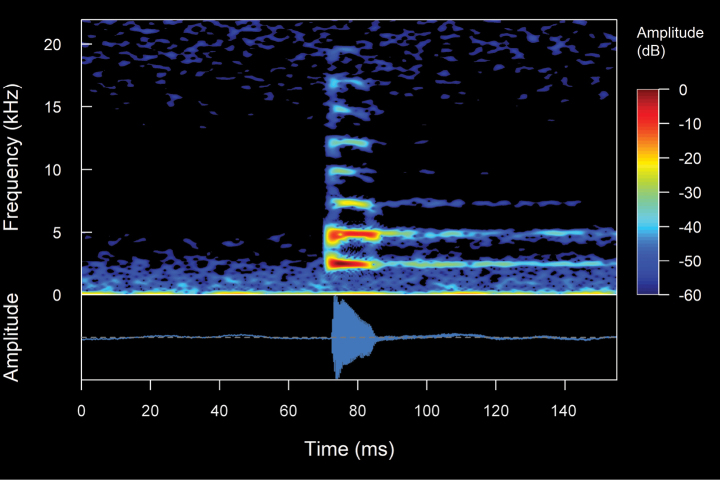
Spectrogram and oscillogram of the advertisement call of *Pristimantis* sp. 4 *myersi* group (ZSFQ 4427, SVL 15.28 mm, 7.9 °C air temperature, 82% relative humidity). Spectrogram obtained using the Hann window at 99% overlap, 256 samples of FFT size and 3 dB filter bandwidth of 248 Hz.

We recorded six males (Suppl. material 2, Table 2). The call (Fig. 24) consists of the emission of a non-pulsed note. Their sounds are like a kind of metallic tapping that onomatopoeically resembles a “tic”. The recorded males were calling from dense undergrowth, leaf litter, within hollow trunks, and the base and interior of adventitious tree roots. The calls are of medium to high intensities and they can be audible at distances of up to ca 100 m. This is a crepuscular and nocturnal species (with little diurnal activity), with moderate to high vocal activity, which intensifies after a rain and in response to the calls of nearby males. The mean call duration is 149.16 ± 251.15 ms (range 9–903 ms), emitted at mean intervals of 5.21 ± 0.87 s (range 3.85–9.15 s), with a mean rate of 11.45 ± 1.91 calls/minute (range 5.91–15.54 calls/minute). The calls are composed of a mean of 1.24 ± 0.43 notes (range 1–2 notes). The mean note duration is 11.88 ± 1.07 ms (range 10–13 ms), emitted at mean intervals of 567.93 ± 94.17 ms (range 467–877 ms), with a mean rate of 1.76 ± 0.23 notes/second (range 1.12–2.08 notes/second). The calls are downward frequency modulated (upward in some calls), with a mean frequency modulation of 14.68 ± 8.7 Hz/ms (range 0–35.83 Hz/ms). The mean dominant frequency (coincides with the fundamental) is 2.57 ± 0.08 kHz (range 2.33–2.76 kHz). The mean minimum frequency is 2.36 ± 0.09 kHz (range 2.15–2.5 kHz), while the mean maximum frequency is 2.76 ± 0.08 kHz (range 2.58–2.93 kHz). Up to eight harmonics are visible, the second having a mean frequency of 5.13 ± 0.19 kHz (range 4.65–5.51 kHz) and the eighth having a mean frequency of 20.07 ± 0.5 kHz (range 19.29–20.76 kHz).

#### *Pristimantis* sp. 5 (*Pristimantismyersi* species group)

**Figure 25.**
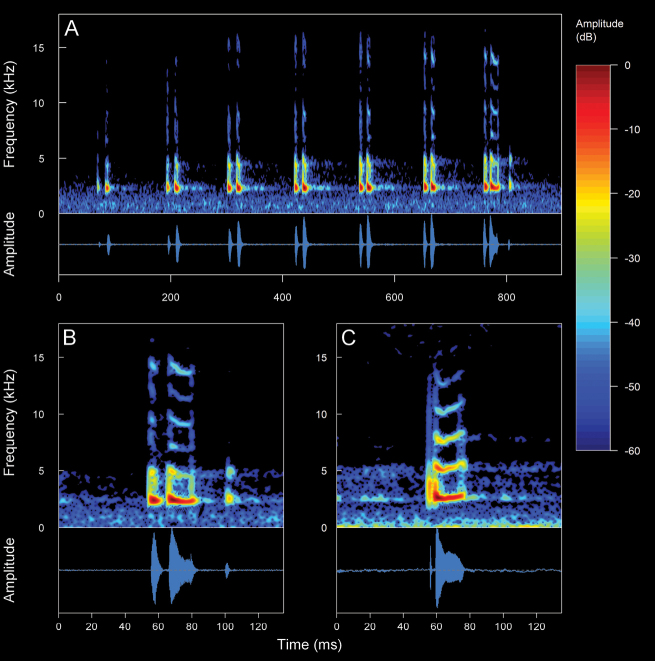
Spectrograms and oscillograms of the advertisement call of *Pristimantis* sp. 5 *myersi* group. **A** complete advertisement call **B** detail of a pulsed note with downward frequency modulation (**A, B**DHMECN 13334, SVL 17.1 mm, 8.3 °C air temperature, 78% relative humidity) **C** detail of a pulsed note with upward frequency modulation (ZSFQ 4500, SVL 18.27 mm, 11.8 °C air temperature, 93% relative humidity). Spectrograms obtained using the Hann window at 99% overlap, 256 samples of FFT size and 3 dB filter bandwidth of 248 Hz.

We recorded six males (Suppl. material 2, Table 2). The call (Fig. 25) consists of the continuous emission of pulsed notes. The recorded males were calling from grassland, leaf litter, and shrubby vegetation between 40 and 80 cm above the ground. The calls are of medium intensity and can be audible at distances particularly in open fields. This species is crepuscular and nocturnal, with moderate vocal activity, which intensifies after a rain and in response to the calls of nearby males. The mean call duration is 357–827 s (range 502.45 ± 123.07 s), emitted at mean intervals of 22.26 ± 13.55 s (range 2.89–57.66 s) with a mean rate of 3.96 ± 3.18 calls/minute (range 1.03–18.13 calls/minute). This species usually emits two to three consecutive calls grouped in series, at mean intervals of 538.33 ± 50.8 ms (range 429–616 ms). The calls are composed of a mean of 6.85 ± 0.96 notes (range 5–9 notes). The mean note duration is 20.88 ± 7.29 ms (range 2–48 ms), emitted at mean intervals of 61.34 ± 12.7 ms (range 40–108 ms), with a mean rate of 12.58 ± 1.76 notes/second (range 8.13–19.23 notes/second). The notes are composed of a mean of 3.02 ± 1.06 pulses (range 1–7 pulses). The mean pulse duration is 5 ± 2.61 ms (range 1–21 ms), the last pulse being the longest of the call, with a mean duration of 12.79 ± 3.83 ms (range 7–21 ms). The pulses are emitted at mean intervals of 3.45 ± 3.01 ms (range 0.5–25 ms), with a mean rate of 185.46 ± 100.59 pulses/second (range 14.08–500 pulses/second). The calls are non-frequency modulated, exhibiting upward-downward modulation in their notes (8.01 ± 5.7 Hz/ms). The mean dominant frequency (coincides with the fundamental) is 2.48 ± 0.14 kHz (range 2.15–2.84 kHz). The mean minimum frequency is 2.22 ± 0.16 kHz (range 1.72–2.58 kHz), while the mean maximum frequency is 2.77 ± 0.19 kHz (range 2.41–3.62 kHz). Up to eight harmonics are visible.

## Discussion

The present study describes the spectral and temporal parameters of advertisement calls of 20 anurans from the high Andean ecosystems of northern Ecuador, Carchi province. Only the calls of five of these species have already been reported in previous studies (Table 1). Sinsch and Juraske (2006) describe the call of *Gastrothecaorophylax*, reported from a locality in the high Andean ecosystems of northern Ecuador (San Gabriel, Carchi). The authors mention that the calls of *G.orophylax* are pulsed, with a mean duration of 2.47 ± 0.42 s (3.01 ± 1.47 s in this study) and a mean dominant frequency of 0.99 ± 0.1 kHz (0.98 ± 0.12 kHz in this study). This indicates spectral values that are similar to those described in the present study, although with different temporal values. In this regard, the variation of temporal parameters in anuran calls may be associated with environmental and social factors, such as temperature, specific habitats, and behavioral strategies (Zimmerman 1983; Jansen et al. 2016; Muñoz et al. 2020; Gillard and Rowley 2023; Mendoza-Henao et al. 2023). *Centrolenebuckleyi* is the species with the highest number of call descriptions in Ecuador and Colombia. Consequently, it is the best studied species in the northern Andes (e.g., Guayasamin et al. 2006; Almendáriz and Batallas 2012; Duarte-Marín et al. 2022). It has been proposed that *C.buckleyi* is a species complex, as evidenced by molecular data (Amador et al. 2018; Guayasamin et al. 2020; Cisneros-Heredia et al. 2023; Cuellar-Valencia et al. 2023; Franco-Mena et al. 2024). However, the calls exhibit structural similarities, characterized by short pulses, upward frequency modulation, and a dominant frequency not exceeding 3.3 kHz. Despite these similarities, the complex has yet to be evaluated with acoustic evidence at the level of its variation.

The call characteristics of *Pristimantisfestae* are consistent with those presented by Holzheuser and Merino-Viteri (2019) and recorded at Hacienda Zuleta, Imbabura province, Ecuador. They agree that the calls are high-pitched whistles composed of one or two notes. Holzheuser and Merino-Viteri (2019) reported that calls of *P.festae* are composed of one or two short auxiliary notes that second a main note. The present study, the call of *P.festae* contains more than two auxiliary elements, which we interpret and consider to be structural pulses of a note. In addition to the designation of these elements as notes or pulses, we concur that a correlation may exist between these elements and a potential behavioral or environmental context that could result in variation in the structure of the call. In terms of activity, the recordings obtained by Holzheuser and Merino-Viteri (2019), were made during the day, with no nocturnal recordings. This contrasts with the data obtained in the present study, where the highest vocal activity was nocturnal and all recordings of *P.festae* were obtained at night.

Another previously reported call is that of *P.buckleyi*, as described by Cuellar-Valencia et al. (2023), from individuals recorded in southwestern Colombia. The mean duration of the call was reported to be 56 ± 4 ms (51.46 ± 4.77 ms in the present study), with a mean call rate of 15.007 ± 0.652 calls/minute (15.12 ± 5.63 calls/minute in the present study), while the mean dominant frequency is 1.01 ± 0.02 kHz (1.26 ± 0.07 kHz in the present study). As with *Centrolenebuckleyi*, the calls of *Pristimantisfestae* and *Pristimantisbuckleyi* described in this study are similar to those previously documented. The subtle spectral and temporal variations in their calls may have taxonomic implications, aligning with molecular evidence that suggests these species form a species complex (Franco-Mena et al. 2023; Reyes-Puig et al. 2023).

The major contribution of the current study is the first description of vocalizations from 15 species, ten of which belong to the genus *Pristimantis*, a genus with an outstanding richness (613 species described so far; Frost 2024), but whose the bioacoustic, ecological, and ethological information remain scarce (Batallas and Brito 2016; Hutter et al. 2016). It is likely that this limited knowledge is a consequence of short-term studies based only on direct visual encounters (Batallas and Brito 2023), as well as the difficulties associated obtaining high-quality recording in harsh environments such as the Andean highlands at night. It is also important to note that the lack of bioacoustical information is a wide reality for most anuran species (De la Riva et al. 1995; Márquez et al. 1995; Köhler et al. 2017).

Notably, in the present study we describe the call of *Niceforoniabrunnea*, a species that is characterized by the absence of vocal slits and sacs. The description of this call contributes to the growing body of evidence indicating that species that lack these structures and have historically been considered mute may in fact possess the capacity to vocalize (see Duellman 1978; Batallas and Brito 2022).

This study also presents the first description of the call of *Hyloxalusdelatorreae*, a species endemic to the province of Carchi-Ecuador and critically endangered (Coloma et al. 2022). In a study conducted by Yánez-Muñoz et al. (2010), the population status of *H.delatorreae* was evaluated in the same area where the call of this study was recorded (Moran-Carchi). The study estimated there to be a number of 52 individuals in an area of ca 5 ha. The population was found to be facing a number of critical problems, and the possibility of extinction is predicted. It is noteworthy that *H.delatorreae* has not been recorded again since February 2017, suggesting the local, and most likely global, extinction of this endemic species.

This is the first study to provide a general view of the acoustic diversity of anurans inhabiting the high Andean ecosystems of northern Ecuador. These ecosystems remain poorly understood and are significantly impacted by human activities (Yánez-Muñoz et al. 2020). The confirmation of nine candidate species and the southernmost record of *Pristimantisfarisorum* underscores the necessity of implementing supplementary studies employing both active and passive methodologies (e.g., passive acoustic monitoring with programmable audio recorders) in these fragile and vulnerable ecosystems. In addition, the groupings obtained in the results of the Principal Component Analysis (PCA), according to the parameters analyzed, demonstrate the diversity and acoustic identity. Therefore, the importance of the use of acoustic parameters in the classification and identification of anuran species is supported and confirmed in a preliminary and prospective way Complementing acoustic data with molecular phylogenies and morphology will leads us to a better and deeper understanding of the anurofauna, with the aim of conserving the invaluable high Andean ecosystems.

## ﻿Additional information

### Author ORCIDs

Diego Batallas https://orcid.org/0000-0002-0068-8146

Rafael Márquez https://orcid.org/0000-0002-2070-860X

Juan M. Guayasamin https://orcid.org/0000-0003-0098-978X

### Data availability

All of the data that support the findings of this study are available in the main text or Supplementary Information.

## Supplementary materials

Supplementary material 1 Anurans of Carchi-Ecuador Data type xlsx Explanation note This database contains collection data and recordings of anurans from the high Andean ecosystems of Carchi, Ecuador (2016–2021). https://binary.pensoft.net/file/1238856This dataset is made available under the Open Database License (http://opendatacommons.org/licenses/odbl/1.0/). The Open Database License (ODbL) is a license agreement intended to allow users to freely share, modify, and use this Dataset while maintaining this same freedom for others, provided that the original source and author(s) are credited.Diego Batallas, Rafael Márquez, Juan M. Guayasamin

Supplementary material 2 Bioacoustics of Anurans from Carchi, Ecuador Data type xlsx Explanation note This database contains the acoustic measurements obtained from the calls of the various specimens collected and not collected in the study. It details the sample number in relation to each specimen, the calls, the notes, and the pulses. https://binary.pensoft.net/file/1238857This dataset is made available under the Open Database License (http://opendatacommons.org/licenses/odbl/1.0/). The Open Database License (ODbL) is a license agreement intended to allow users to freely share, modify, and use this Dataset while maintaining this same freedom for others, provided that the original source and author(s) are credited.Diego Batallas, Rafael Márquez, Juan M. Guayasamin
